# Reduced mTORC1-signalling in retinal progenitor cells leads to visual pathway dysfunction

**DOI:** 10.1242/bio.044370

**Published:** 2019-07-08

**Authors:** Iwan Jones, Anna-Carin Hägglund, Leif Carlsson

**Affiliations:** Umeå Center for Molecular Medicine (UCMM), Umeå University, 901 87 Umeå, Sweden

**Keywords:** Raptor, mTORC1, Retina, RGCs, dLGN, Visual cliff test

## Abstract

Development of the vertebrate central nervous system involves the co-ordinated differentiation of progenitor cells and the establishment of functional neural networks. This neurogenic process is driven by both intracellular and extracellular cues that converge on the mammalian target of rapamycin complex 1 (mTORC1). Here we demonstrate that mTORC1-signalling mediates multi-faceted roles during central nervous system development using the mouse retina as a model system. Downregulation of mTORC1-signalling in retinal progenitor cells by conditional ablation of *Rptor* leads to proliferation deficits and an over-production of retinal ganglion cells during embryonic development. In contrast, reduced mTORC1-signalling in postnatal animals leads to temporal deviations in programmed cell death and the consequent production of asymmetric retinal ganglion cell mosaics and associated loss of axonal termination topographies in the dorsal lateral geniculate nucleus of adult mice. In combination these developmental defects induce visually mediated behavioural deficits. These collective observations demonstrate that mTORC1-signalling mediates critical roles during visual pathway development and function.

## Abstract (precis)

**Summary:** Conditional deletion of *Rptor* in retinal progenitor cells demonstrates that mTORC1-signalling is critical for visual pathway development and function.

## INTRODUCTION

**Fig. 1.**
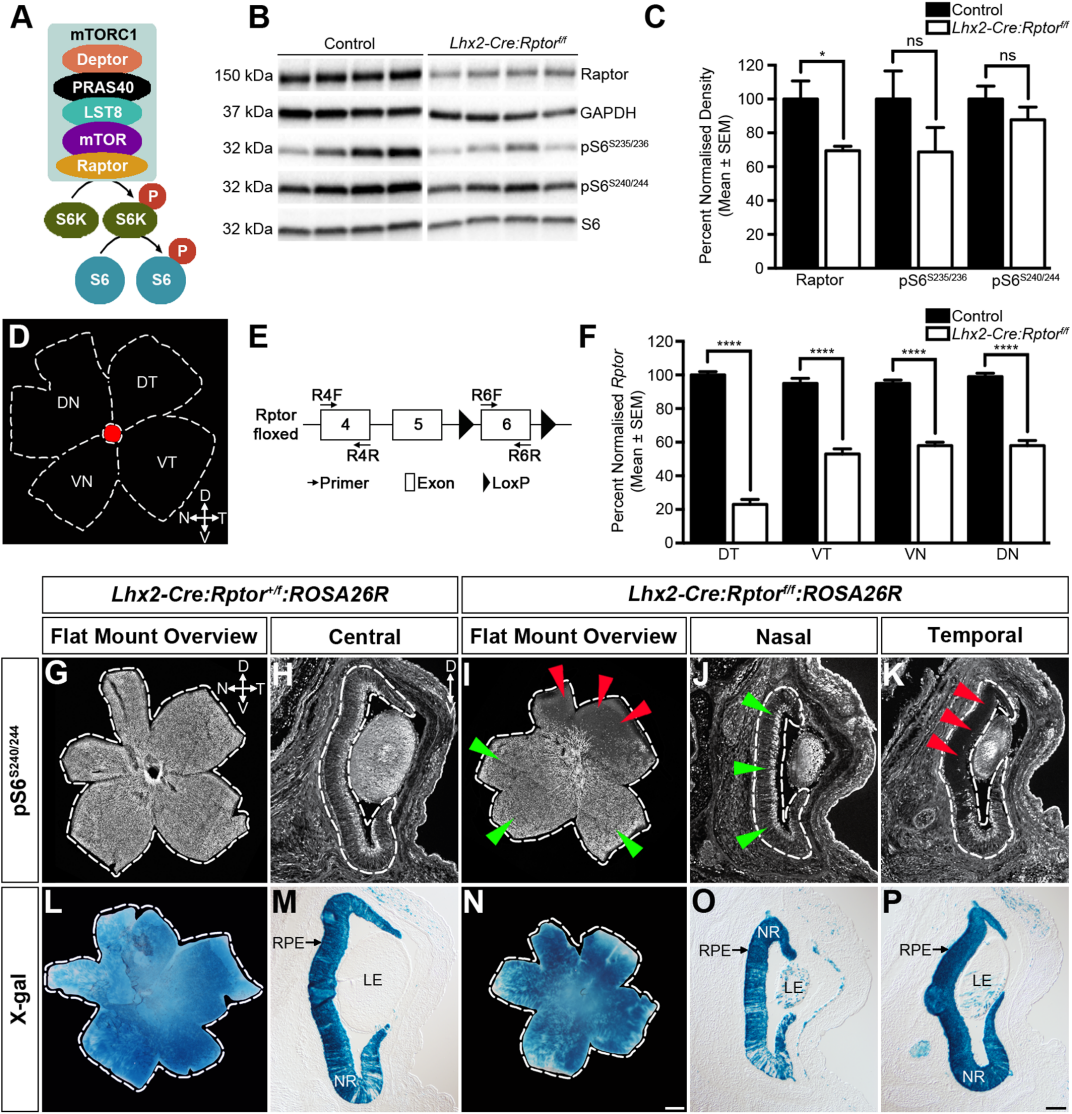
**Conditional deletion of *Rptor* leads to domain-specific reductions in mTORC1-signalling.** (A) The mTORC1 is assembled from Deptor, PRAS40, LST8, mTOR and Raptor. This multimeric complex regulates protein translation in response to nutritional and environmental cues through the phosphorylation of the 40S ribosomal protein S6 (pS6). (B) Representative immunoblot analyses of Raptor, GAPDH, pS6^S235/236^, pS6^S240/244^ and S6 in retinal extracts harvested from control (*n*=4) and *Lhx2-Cre:Tsc1^f/^*^f^ (*n*=4) mice at P1. (C) Densitometry analyses of Raptor, pS6^S235/236^ and pS6^S240/244^ in retinal extracts harvested from control (*n*=6) and *Lhx2-Cre:Rptor^f/f^* (*n*=6) mice at P1. *Lhx2-Cre:Rptor^f/f^* mice exhibit a significant 30% decrease in Raptor levels compared to littermate controls. No significant differences in pS6^S235/236^ and pS6^S240/244^ levels were observed. Raptor densitometry data were normalised against GAPDH while pS6^S235/236^ and pS6^S240/244^ values were normalised against S6. (D) Schematic diagram detailing the dissection strategy employed for harvesting retinal quadrants. (E) Schematic diagram detailing the qPCR strategy employed to determine the level of *Rptor* recombination in the retinal quadrants. The level of PCR product generated by the target primer pair (R6F and R6R) was normalised against that amplified from the internal control primer pair (R4F and R4R). (F) qPCR analysis of normalised *Rptor* levels in retinae harvested from control (*n*=6) and *Lhx2-Cre:Rptor^f/f^* (*n*=7) mice at 6 weeks of age. Mutant animals exhibit a domain-specific pattern of *Rptor* recombination with approximately 80% deletion being observed in the DT retina while the remaining quadrants exhibit close to a 50% deletion rate. (G–K) Representative immunohistochemical staining of retinae harvested from *Lhx2-Cre:Rptor^+/f^:ROSA26R* (G–H) and *Lhx2-Cre:Rptor^f/f^:ROSA26R* (I–K) mice at P1. *Lhx2-Cre:Rptor^+/f^:ROSA26R* animals exhibit widespread pS6^S240/244^ distribution due to canonical mTORC1-signalling mediated by the remaining wild-type *Rptor* allele. In contrast, no pS6^S240/244^ was observed in the DT retina (I,K, red triangles) of *Lhx2-Cre:Rptor^f/f^:ROSA26R* mice while modest pS6^S240/244^ levels were observed within the remaining regions (I,J, green triangles). (L–P) Representative lineage tracing analyses of retinae taken from *Lhx2-Cre:Rptor^+/f^:ROSA26R* (L,M) and *Lhx2-Cre:Rptor^f/f^:ROSA26R* (N–P) mice at P1. All mice exhibit widespread X-gal staining throughout the retina thus confirming global *Lhx2-Cre* transgene expression and subsequent Cre-mediated recombination of the *ROSA26R* allele. Note that radial columns of X-gal mosaicism are observed in both *Lhx2-Cre:Rptor^+/f^:ROSA26R* and *Lhx2-Cre:Rptor^f/f^:ROSA26R* mice due to variable *ROSA26R* recombination in the ventral retina (M,O). The broken line demarcates the border of the retina in all images. All data represents the mean±s.e.m. Statistical differences were calculated using unpaired two-tailed Student's *t*-tests. *P*-values are denoted as follows: *P≤0.05 and ****P≤0.0001. Scale bars: (G,I,L,N) 500 µm; (H,J,K,M,O,P) 100 µm. Abbreviations: D, dorsal; Deptor, DEP domain containing mTOR interacting protein; DN, dorsonasal; DT, dorsotemporal; GAPDH, glyceraldehyde 3-phosphate dehydrogenase; kDa, kilodalton; LE, lens; LST8, lethal with SEC13 protein 8; mTOR, mechanistic target of rapamycin; mTORC1, mechanistic target of rapamycin complex 1; N, nasal; NR, neural retina; P, postnatal day; PRAS40, proline rich AKT substrate 40 kDa; pS6, phosphorylated ribosomal protein S6; pS6K, phosphorylated ribosomal protein S6 kinase; Raptor, regulatory protein associated with mTOR; RPE, retinal pigment epithelium; S6, ribosomal protein S6; S6K, ribosomal protein S6 kinase; T, temporal; V, ventral; VN, ventronasal; VT, ventrotemporal; X-gal, 5-bromo-4-chloro-3-indolyl-β-D-galactopyranoside.

Development of the vertebrate central nervous system (CNS) involves the transition of neural progenitor cells (NPCs) through a series of temporal and spatial neurogenic waves that lead to the establishment of functional neural networks comprised of distinct cellular lineages (Götz and Huttner, 2005). Neurogenesis is driven by both intracellular and extracellular molecules that signal through the mammalian target of rapamycin complex 1 (mTORC1) to coordinate cell growth, nutritional metabolism and protein translation in response to these inductive cues (LiCausi and Hartman, 2018). mTORC1 is a protein complex that is assembled from three regulatory (mTOR, Raptor and mLST8) and two inhibitory components (PRAS40 and Deptor) (Saxton and Sabatini, 2017) (Fig. 1A). Raptor functions as an adaptor protein and is responsible for recruiting canonical mTORC1 substrates through binding to their TOR signalling motifs (Nojima et al., 2003). One downstream effector is p70S6K whose phosphorylation of the 40S ribosomal protein S6 (pS6) routinely acts as a readout marker of mTORC1 signalling (Holz et al., 2005; Knight et al., 2012) (Fig. 1A).

The generation of Raptor deficient animal models has been a robust approach for demonstrating that mTORC1-signalling is critical for the development and function of several major cell types within the CNS. Conditional deletion of *Rptor* in NPCs during embryonic development leads to microcephaly and aberrant oligodendrocyte differentiation while *Rptor* inactivation in postnatal mice induces reactive astrogliosis (Bercury et al., 2014; Chen et al., 2016; Cloetta et al., 2013; Zhang et al., 2017). Furthermore, treatment of experimental models with pharmacological mTORC1 inhibitors leads to long-term learning and memory deficits (Blundell et al., 2008; Tischmeyer et al., 2003). Taken together, these observations demonstrate that mTORC1-signalling is integral to brain development and function but whether this pathway mediates similar multifaceted roles within other CNS domains remains largely unexplored.

The mouse retina is a tractable model for studying various aspects of CNS development and function (Agathocleous and Harris, 2009). This neuroepithelial tissue develops from a homogenous pool of retinal progenitor cells (RPCs) that undergo orchestrated cell fate transitions to produce six neuronal classes: rod and cone photoreceptors, horizontal, bipolar, amacrine and retinal ganglion cells (RGCs) and Müller glia. The adult mouse retina presents as a laminated structure with the cell bodies of these distinct cell lineages being radially positioned within three stratified layers: (i) photoreceptors reside in the outer nuclear layer (ONL), (ii) the inner nuclear layer (INL) contains horizontal, bipolar and amacrine cells in addition to Müller glia, while the (iii) ganglion cell layer (GCL) contains RGCs and displaced amacrine cells (Bassett and Wallace, 2012). Within these stratified layers various neuronal classes are also tangentially distributed across the retinal surface in well-ordered mosaics to ensure complete sampling of visual space. Of particular importance in this regard are RGC mosaics whose primary function is to relay visual information to retinorecipient brain nuclei such as the dorsal lateral geniculate nucleus (dLGN) (Morin and Studholme, 2014; Reese and Keeley, 2015). This precise tissue architecture requires both intracellular and extracellular signalling (Amini et al., 2017) and recent studies have demonstrated the importance of the mTORC1 pathway for radial positioning during mouse retinal development (Choi et al., 2018; Jones et al., 2015). However, whether mTORC1 mediates similar roles during tangential mosaic formation and visually mediated behaviour is currently unknown.

The work presented in this study therefore addresses the roles of mTORC1-signalling during retinal mosaic formation. We demonstrate that downregulation of this pathway in RPCs by means of *Rptor*-ablation induces proliferation deficits and aberrant RGC differentiation during mouse embryonic development. Such neurogenic disruption consequently leads to asymmetric RGC mosaics and irregular binocular projection topographies in the dLGN of adult animals. These multi-faceted developmental defects eventually culminate in functional deficits when performing visually mediated behavioural tasks. Our collective observations therefore demonstrate that mTORC1-signalling mediates critical roles during various aspects of retinal mosaic formation and visual pathway function.

## RESULTS

### Conditional deletion of *Rptor* within retinal progenitor cells leads to a distinct spatial pattern of reduced mTORC1-signalling

To disrupt mTORC1-signalling in RPCs (Fig. 1A) we crossed mice carrying floxed *Rptor* alleles (*Rptor^tm1.1Dmsa^*, referred to as *Rptor^f/f^*) (Sengupta et al., 2010) with animals harbouring an *Lhx2*-promoter driven Cre-recombinase transgene (*Tg(Lhx2-Cre)1Lcar*, referred to as *Lhx2-Cre*) (Hagglund et al., 2011), which promotes *LoxP* recombination in RPCs prior to the onset of neurogenesis and mTORC1-signalling (Hagglund et al., 2011, 2017). *Lhx2-Cre:Rptor^f/f^* mice were born at normal Mendelian ratios and adult mice were indistinguishable from their littermates (Fig. S1). Immunoblot analyses were therefore performed on retinal extracts harvested at postnatal day 1 (P1) to determine the extent of mTORC1 pathway activity following *Rptor* deletion. While a significant reduction in Raptor was detected in *Lhx2-Cre:Rptor^f/f^* animals, the level of pS6 was comparable to control littermates (Fig. 1B,C). One possible explanation for this apparent normal level of mTORC1-signalling was that the *Rptor* allele was undergoing variable recombination efficiency within the RPC population. Retinae were therefore harvested from control and *Lhx2-Cre:Rptor^f/f^* adult mice and dissected into four equal tissue quadrants (Fig. 1D). Genomic DNA was prepared from these regions and the level of *Rptor* recombination was determined by qPCR (Fig. 1E). *Lhx2-Cre:Rptor^f/f^* animals exhibited a distinct spatial pattern of *Rptor*-ablation. A recombination level of approximately 80% was detected in the dorsotemporal (DT) region while the remaining retina exhibited close to a 50% deletion rate (Fig. 1F).

Given the distinct spatial pattern of *Rptor* recombination we predicted that the DT retina of *Lhx2-Cre:Rptor^f/f^* mice would exhibit a lower level of mTORC1-signalling relative to the remaining quadrants. Immunohistochemical analyses were therefore performed on flat-mounted retinae harvested from *Lhx2-Cre:Rptor^+/f^:ROSA26R* and *Lhx2-Cre:Rptor^f/f^:ROSA26R* animals at P1 (Fig. 1G–K). Widespread pS6^S40/244^ expression was observed throughout the retina of *Lhx2-Cre:Rptor^+/f^:ROSA26R* mice due to canonical mTORC1-signalling mediated by the remaining wild-type allele (Fig. 1G,H). In contrast, no pS6^S240/244^ was detected in the DT retina of *Lhx2-Cre:Rptor^f/f^:ROSA26R* mice (Fig. 1I,K, red triangles) in combination with normal pS6^S240/244^ levels being observed within the remaining quadrants (Fig. 1I,J, green triangles). Importantly, this distinct spatial pattern of *Rptor* recombination was not due to variable expression of the *Lhx2-Cre* transgene since lineage-tracing analyses demonstrated widespread X-gal staining throughout the retina of both *Lhx2-Cre:Rptor^+/f^:ROSA26R* and *Lhx2-Cre:Rptor^f/f^:ROSA26R* mice (Fig. 1L–P). However, it should be noted that radial columns of X-gal mosaicism was sometimes observed in both *Lhx2-Cre:Rptor^+/f^:ROSA26R* and *Lhx2-Cre:Rptor^f/f^:ROSA26R* mice due to subsets of RPCs escaping *ROSA26R* recombination in the ventral retina (Fig. 1M,O).

To summarise, a distinct spatial pattern of *Rptor*-ablation was observed in *Lhx2-Cre:Rptor^f/f^* mice (Fig. S2). RPCs that generated the DT retina exhibited high levels of deletion that consequently resulted in the loss of mTORC1-signalling within this domain. In contrast, progenitor cells that established the remaining retina exhibited lower levels of *Rptor* recombination that had no outward effects on mTORC1-signalling activity. This characteristic spatial pattern of *Rptor*-ablation was also fully penetrant amongst all *Lhx2-Cre:Rptor^f/f^* mice analysed (*n*=43) independent of sex or genetic background (*129/Sv:CBA:C57BL/6* and *129/Sv:CBA:C57BL/6:NMRI*).

### Domain-specific reduction in mTORC1-signalling leads to aberrant RGC differentiation during embryonic development

**Fig. 2.**
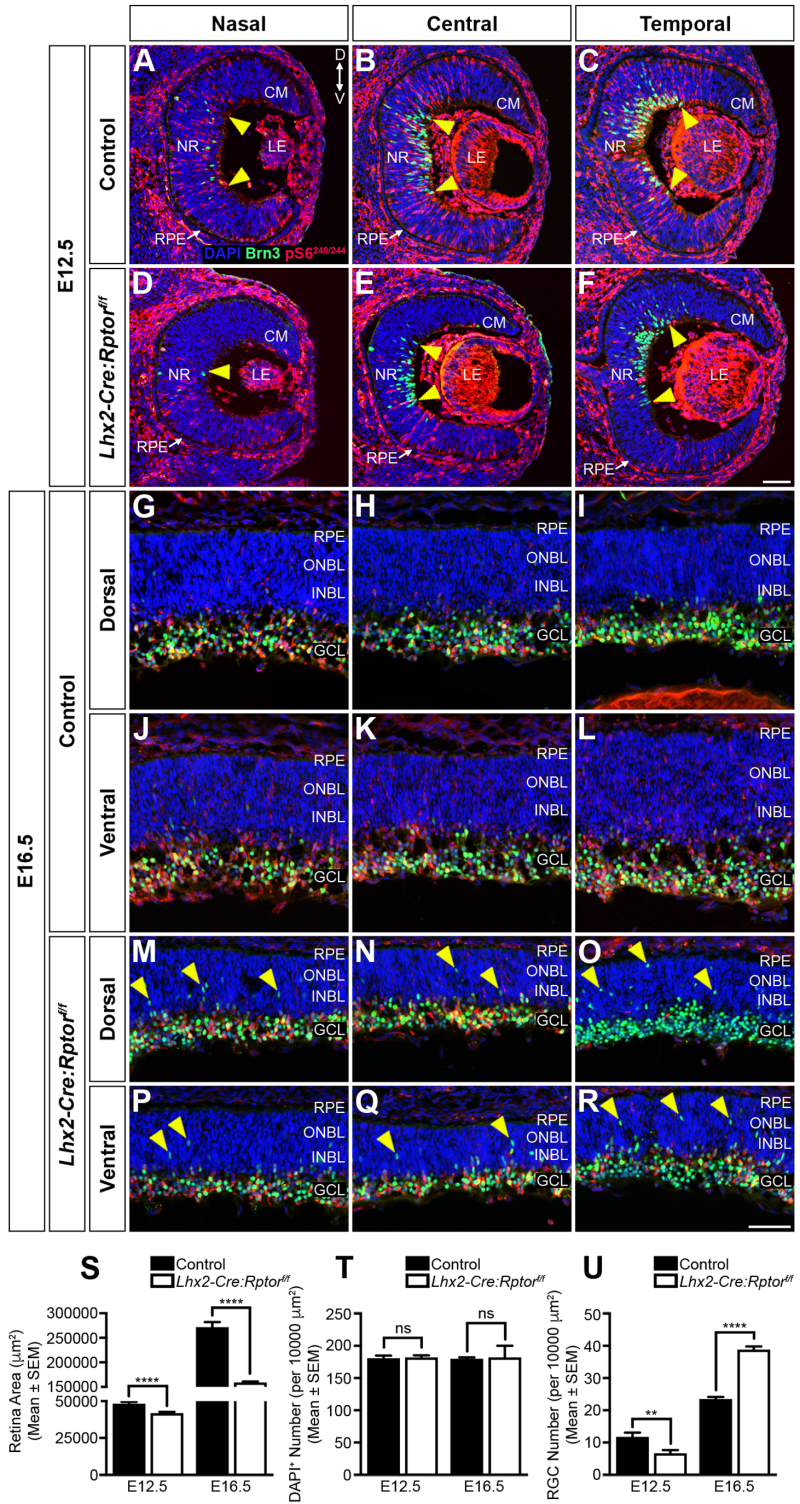
**Domain-specific reduction in mTORC1-signalling leads to aberrant RGC neurogenesis and radial migration deficits during embryonic development.** RGC differentiation and mTORC1-activity was assessed by immunohistochemistry at developmental time points that coincided with neurogenic onset (E12.5) and radial migration (E16.5) milestones. (A–F) Representative coronal sections through the nasal to temporal axis of control (A–C) and mutant (D–F) animals at E12.5 demonstrates the delayed appearance of RGCs and reduced mTORC1-signalling in *Lhx2-Cre:Rptor^f/f^* mice. The yellow triangles represent the dorsal and ventral boundaries of the differentiating Brn3^+^ RGC population, respectively. Note that both control and mutant animals display similar nasal^low^ to temporal^high^ RGC distribution gradients. (G–R) Representative coronal sections through the nasal to temporal axis of control (G–L) and *Lhx2-Cre:Rptor^f/f^* (M–R) animals at E16.5 reveals ectopic Brn3^+^ cells (yellow arrowheads) in both the dorsal and ventral neuroblastic layers of mutant mice. Moreover, a reduced level of mTORC1-signalling is observed throughout the whole extent of the retina in *Lhx2-Cre:Rptor^f/f^* animals with the DT region (O) exhibiting a complete lack of pS6^S240/244^ immunoreactivity. (S) Quantitative analysis demonstrates that the area of the retina in *Lhx2-Cre:Rptor^f/f^* mice was significantly smaller during both neurogenesis (E12.5) and radial migration (E16.5). (T) Quantitative analysis demonstrates that the density of DAPI^+^ cells in the retina of both control and *Lhx2-Cre:Rptor^f/f^* mice were comparable during both neurogenesis (E12.5) and radial migration (E16.5). (U) Quantitative analysis demonstrates that the density of Brn3^+^ cells in the retina of *Lhx2-Cre:Rptor^f/f^* mice was significantly reduced during neurogenesis (E12.5) but the subsequent over-production of RGCs leads to a significant increase in their numbers during radial migration (E16.5). The number of eyes analysed at each age was as follows: E12.5 (control, *n*=8; *Lhx2-Cre:Rptor^f/f^*, *n*=8); E16.5 (control, *n*=8; *Lhx2-Cre:Rptor^f/f^*, *n*=12). All data represents the mean±s.e.m. Statistical differences were calculated using unpaired two-tailed Student's *t*-tests. *P*-values are denoted as follows: **P≤0.01 and ****P≤0.0001. Scale bar: (A–F) 50 µm; (G–R) 50 µm. Abbreviations: CM, ciliary margin; D, dorsal; GCL, ganglion cell layer; INBL, inner neuroblastic layer; LE, lens; NR, neural retina; ONBL, outer neuroblastic layer; RPE, retinal pigment epithelium; V, ventral.

The distinct spatial pattern of *Rptor*-ablation observed in *Lhx2-Cre:Rptor^f/f^* mice fortuitously provided an opportunity to investigate how contrasting reductions in mTORC1-signalling influenced RGC neurogenesis [embryonic day (E) 12.5] and radial migration (E16.5) (Fig. 2) (Pan et al., 2005; Reese, 2011). We initially observed that the retinal area of mutant animals was significantly reduced at both E12.5 and E16.5 (Fig. 2S) albeit with cell densities that were comparable to control animals (Fig. 2T). These observations implied that the smaller retina observed within *Lhx2-Cre:Rptor^f/f^* mice was due to a decrease in absolute cell number that presumably arose due to proliferation deficits. To address this possibility the proportion of cells in various phases of the cell cycle were examined at commencement of RGC neurogenesis (E12.5) (Fig. S3). Accordingly, an *in utero* pulse of BrdU followed by phosphohistone-H3 (PH3) immunohistochemistry (Fig. S3A,B) demonstrated that *Lhx2-Cre:Rptor^f/f^* mice had a significant reduction in the number of both proliferating BrdU^+^ and PH3^+^ mitotic RPCs (Fig. S3C). Moreover, the number of double-labelled cells was also reduced which was indicative of an increase in cell cycle length (Fig. S3C). Taken together, our data demonstrated that slower cell cycle progression and a consequent reduction in absolute cell number underlay the decreased retinal area observed in *Lhx2-Cre:Rptor^f/f^* mice.

Widespread pS6^S240/244^ reactivity was detected throughout the entire retina (Fig. S4A–C) and Brn3^+^ RGCs were observed to curve around the central retina of control mice at E12.5 (Fig. 2A–C, yellow arrowheads) with the number of RGCs being lower in the nasal region (Fig. 2A) relative to the temporal domain (Fig. 2C). Contrastingly, the mutant retina exhibited a reduction in both mTORC1-activity (Fig. S4D–F) and RGC numbers (Fig. 2D–F, yellow arrowheads) that was subsequently confirmed by cell count analysis (Fig. 2U). Moreover, the spatial expression pattern of *Math5*, which is essential for RGC differentiation (Yang et al., 2003), overlapped with that of Brn3^+^ cells in the temporal retina of control mice (Fig. S5A) while its expression domain was reduced in mutant animals (Fig. S5B); thus confirming that *Rptor*-ablation led to delayed RGC neurogenesis.

RGC differentiation proceeded in a wave towards the retinal periphery and by E16.5 the majority of Brn3^+^ cells in control mice had migrated into the future GCL (Fig. 2G–L) and their NEFH^+^ axons projected out of the eye to form the optic nerve (Fig. S6A). Moreover, the GCL of wild-type animals exhibited widespread pS6^S240/244^ reactivity (Fig. S7A–F). Contrastingly, a reduction in mTORC1-signalling (Fig. S7G–L) and apicobasal thickness (Fig. S7G–L, brackets) was observed in *Lhx2-Cre:Rptor^f/f^* mice. Interestingly, the mutant animals had an apparent increase in RGC number within the emerging GCL (Fig. 2M–R) that was confirmed by cell count analysis (Fig. 2U) and indirectly by a greater number of projecting NEFH^+^ axons (Fig. S6B). Moreover, multiple ectopic Brn3^+^ cells were also evident within the outer- and inner-neuroblastic layers (Fig. 2M–R, yellow arrowheads). In addition, these phenotypes were most prominent in the DT retina of mutant mice where increased numbers of Brn3^+^ cells (Fig. 2O) and a complete absence of pS6^S240/244^ immunoreactivity (Fig. S7I) were observed. Thus, conditional deletion of *Rptor* in RPCs increased the number of RGCs during embryonic development.

### Domain-specific reduction in mTORC1-signalling leads to altered RGC mosaics during postnatal development

**Fig. 3.**
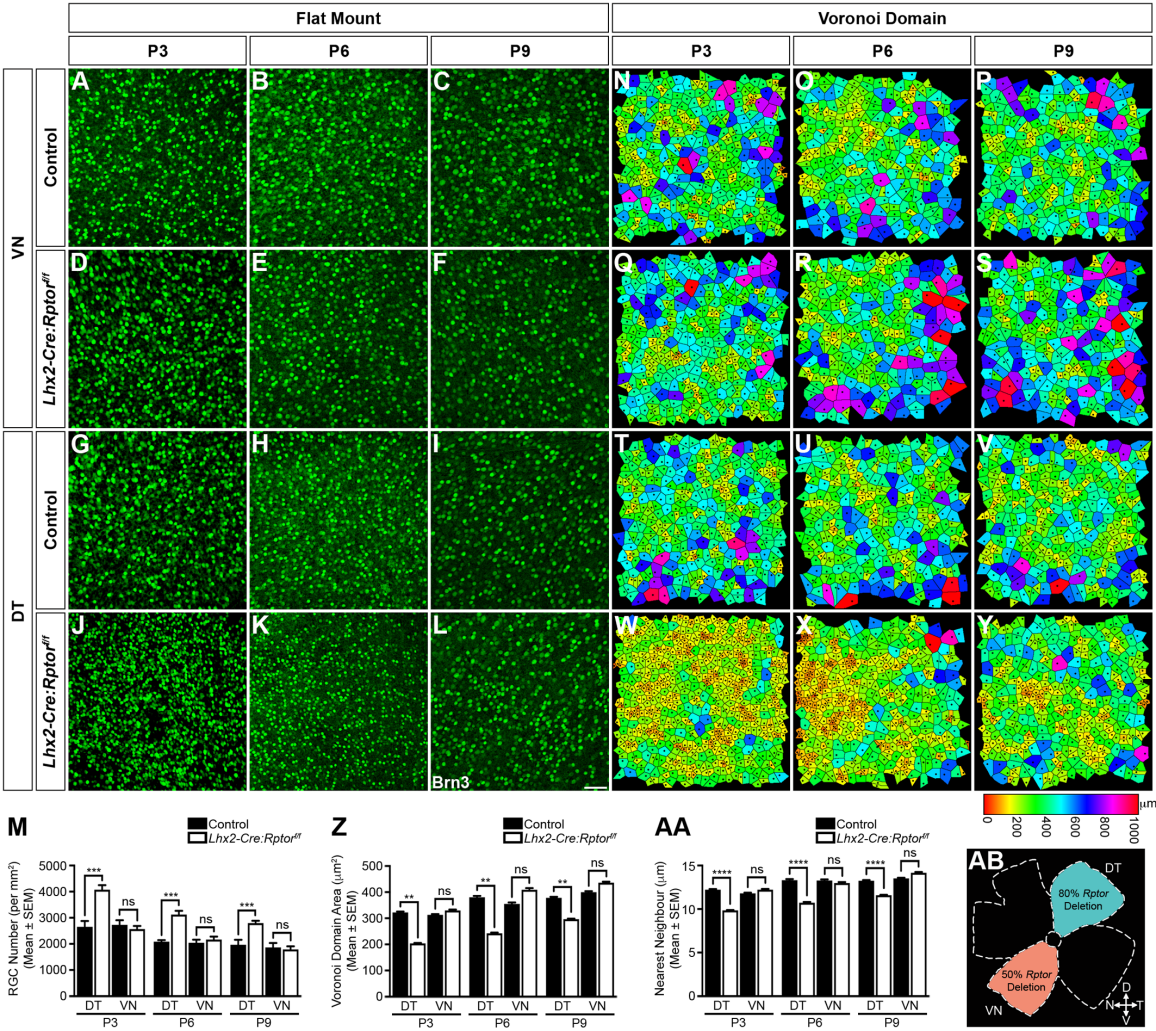
**Domain-specific reduction in mTORC1-signalling leads to an over-production of RGCs in the dorsotemporal retina during postnatal development.** Retinae were harvested from control and *Lhx2-Cre:Rptor^f/f^* mice during postnatal development to determine the mosaic arrangement of RGCs in the VN and DT regions (AB). (A–L) Representative flat-mount analyses of the VN and DT regions of control and *Lhx2-Cre:Rptor^f/f^* mice at P3 (A,D,G,J), P6 (B,E,H,K) and P9 (C,F,I,L) demonstrate that both domains in wild-type animals in addition to the VN region of *Lhx2-Cre:Rptor^f/f^* mice (A–I) exhibit a comparable number of Brn3^+^ cells that are dispersed across the surface of the retina in similar arrays. In contrast, a seemingly greater number of RGCs are present in the DT region of mutant animals at all ages analysed (J–L). (M) Quantification of RGC number in the DT and VN quadrants of control and *Lhx2-Cre:Rptor^f/f^* mice at P3, P6 and P9. Mutant mice have a significant increase in the mean number of Brn3^+^ cells within the DT region at all ages analysed. (N–Y) The regularity of RGC mosaics in the VN and DT regions of control and *Lhx2-Cre:Rptor^f/f^* mice at P3 (N,Q,T,W), P6 (O,R,U,X) and P9 (P,S,V,Y) was quantified by Voronoi domain and nearest neighbour analyses. Representative heat map diagrams reveal comparable RGC territories within both domains of wild-type animals in addition to the VN region of *Lhx2-Cre:Rptor^f/f^* mice (N–V). In contrast, the increased number of RGCs in the DT domain of *Lhx2-Cre:Rptor^f/f^* mice (M) resulted in smaller Voronoi domain areas at all ages analysed (W–Y). (Z,AA) Quantitative analyses of the Voronoi domain (Z) and nearest neighbour distances (AA) demonstrate that RGCs solely within the DT region of *Lhx2-Cre:Rptor^f/f^* mice exhibit significantly reduced Voronoi domain areas and nearest neighbour distances compared to control littermates due to the increased number of cells in this quadrant (M). The corresponding Voronoi domain and nearest neighbour distance histogram plots are presented in Figs S8–S10. (AB) Schematic diagram detailing the retinal quadrants imaged for the Voronoi domain and nearest neighbour analyses. The number of retinae analysed at each age was as follows: P3 (control, *n*=6; *Lhx2-Cre:Rptor^f/f^*, *n*=6); P6 (control, *n*=4; *Lhx2-Cre:Rptor^f/f^*, *n*=4); P9 (control, *n*=11; *Lhx2-Cre:Rptor^f/f^*, *n*=8). All data represents the mean±s.e.m. Statistical differences were calculated using unpaired two-tailed Student's *t*-tests. *P*-values are denoted as follows: **P≤0.01, ***P≤0.001 and ****P≤0.0001. Scale bar: (A–L) 50 µm. Abbreviations: D, dorsal; DT, dorsotemporal; N, nasal; P, postnatal day; T, temporal; V, ventral; VN, ventronasal.

That an over-production of RGCs and radial migration deficits were observed particularly in the DT retina of *Lhx2-Cre:Rptor^f/f^* embryonic mice prompted the assessment of whether *Rptor*-ablation also influenced postnatal RGC mosaic formation (Fig. 3). These analyses were performed in both the DT and ventronasal (VN) quadrants to assess whether the distinct levels of allele recombination observed within these domains influenced tangential dispersion (Fig. 3AB). Flat-mounted retinae taken at postnatal ages (P3, P6 and P9) that coincided with RGC mosaic formation (Reese and Keeley, 2015) demonstrated that both domains in control animals in addition to the VN region of *Lhx2-Cre:Rptor^f/f^* mice exhibited a comparable number of RGCs (Fig. 3A–I). In contrast, a greater number of Brn3^+^ cells were present in the DT retina of mutant animals (Fig. 3J–L) that correlated with the previously observed over-production of Brn3^+^ cells in this domain at E16.5. This apparent increase in DT RGC number was subsequently confirmed by cell count analysis (Fig. 3M).

The mosaic regularity of RGCs residing within the DT and VN retina were next determined by Voronoi domain and nearest neighbour approaches (Galli-Resta et al., 1997; Reese and Keeley, 2015). Both regions in wild-type animals and the VN quadrant of *Lhx2-Cre:Rptor^f/f^* mice (Fig. 3N–V) exhibited comparable Voronoi domain areas. However, the increased number of RGCs in the DT retina of mutant animals resulted in smaller areas at all ages analysed (Fig. 3W–Y), which was confirmed by quantitative analyses (Fig. 3Z,AA). However, no differences in the Voronoi domain (VDRI) or nearest neighbour regularity indices (NNRI) were observed, which demonstrated that control and *Lhx2-Cre:Rptor^f/f^* mice had comparable mosaic symmetries (Figs S8–10).

### Domain-specific reduction in mTORC1-signalling leads to aberrant RGC apoptosis during postnatal development

**Fig. 4.**
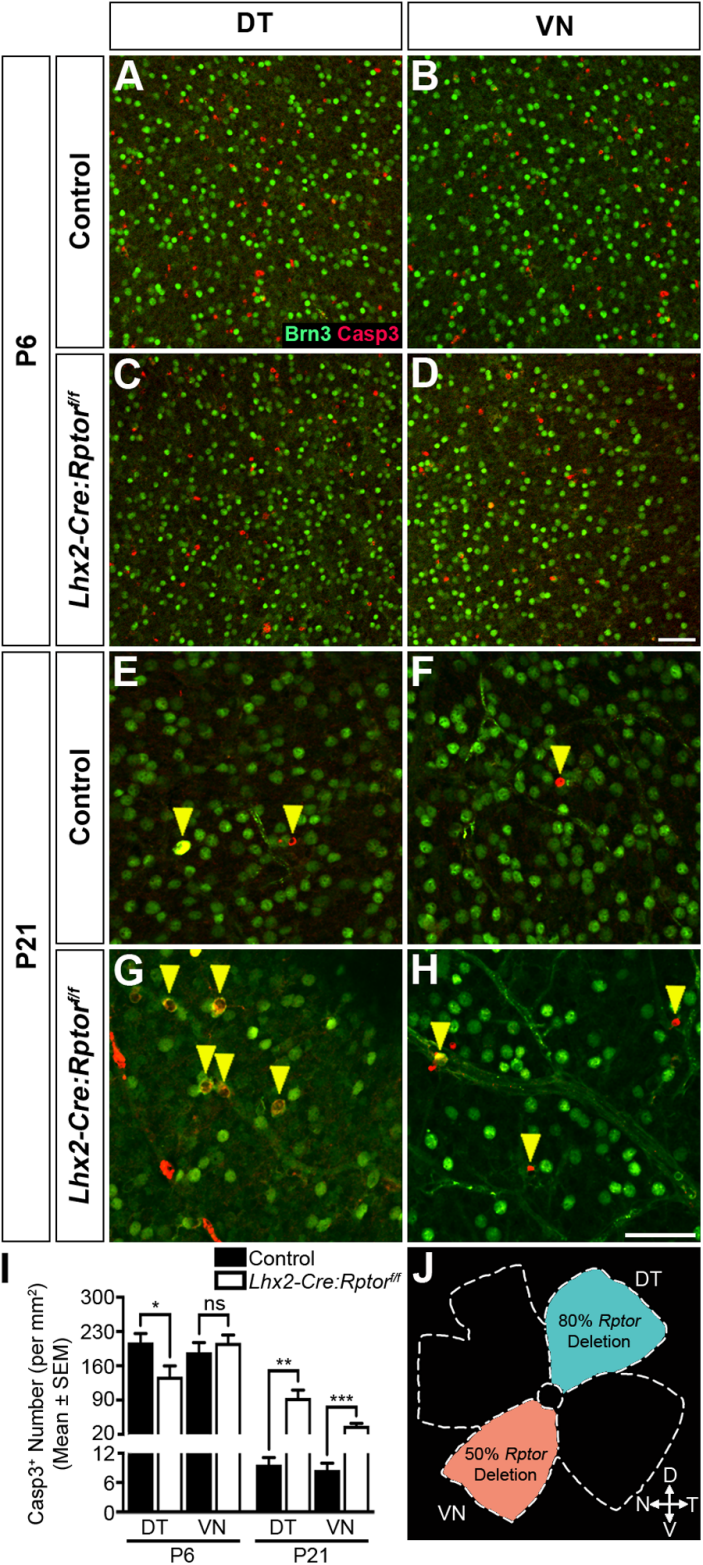
**Domain-specific reduction in mTORC1-signalling leads to aberrant RGC apoptosis during postnatal development.** Retinae were harvested from control and *Lhx2-Cre:Rptor^f/f^* mice during postnatal development to determine the number of apoptotic cells in the DT and VN regions (J). (A–D) Representative flat-mount images of the DT and VN retina in control and *Lhx2-Cre:Rptor^f/f^* mice at P6 demonstrate that both domains in wild-type animals (A–B) in addition to the VN region of *Lhx2-Cre:Rptor^f/f^* (D) mice exhibit a comparable number of Casp3^+^ cells. In contrast, a decreased number of apoptotic cells are observed in the DT region of mutant animals (C). (E–H) Representative flat-mount analyses of the DT and VN retina in control and *Lhx2-Cre:Rptor^f/f^* mice at P21 demonstrate that both domains in wild-type animals (E,F) exhibit solitary Casp3^+^ cells (yellow arrowheads). In contrast, an increased number of apoptotic cells (yellow arrowheads) was observed in both the DT (G) and VN (H) regions of *Lhx2-Cre:Rptor^f/f^* animals. (I) Quantification of Casp3^+^ cell number in the DT and VN quadrants of control and *Lhx2-Cre:Rptor^f/f^* mice. Mutant mice have a significant decrease in the mean number of apoptotic cells within the DT region at P6. Contrastingly, mutant mice have a significant increase in the mean number of apoptotic cells in both the DT and VN region at P21. (J) Schematic diagram detailing the retinal quadrants imaged for the apoptosis analyses. The number of retina analysed at each age was as follows: P6 (control, *n*=12; *Lhx2-Cre:Rptor^f/f^*, *n*=10); P21 (control, *n*=6; *Lhx2-Cre:Rptor^f/f^*, *n*=14). All data represents the mean±s.e.m. Statistical differences were calculated using unpaired two-tailed Student's *t*-tests. *P*-values are denoted as follows: *P≤0.05, **P≤0.01 and ***P≤0.001. Scale bar: (A–D) 50 µm; (E–H) 50 µm. Abbreviations: D, dorsal; DT, dorsotemporal; N, nasal; P, postnatal day; T, temporal; V, ventral; VN, ventronasal.

RGCs undergo extensive postnatal apoptosis to fine-tune their mosaic arrangement (Beros et al., 2018) and we therefore reasoned that alterations in programmed cell death underlay the densely packed nature of RGCs in the DT retina of *Lhx2-Cre:Rptor^f/f^* animals. To address this hypothesis we performed immunohistochemical analyses on flat-mounted retinae harvested from wild-type and mutant mice at P6 (Fig. 4) as this age coincided with the peak of RGC apoptosis during mouse retinal development (Young, 1984). Both DT and VN domains in control (Fig. 4A,B) and the VN region of *Lhx2-Cre:Rptor^f/f^* mice (Fig. 4D) exhibited comparable numbers of evenly dispersed Brn3^+^ nuclei that were intermingled with numerous Casp3^+^ cells. In contrast, densely packed RGCs and a seemingly reduced number of apoptotic cells were present within the DT region of mutant animals (Fig. 4C) that was subsequently confirmed by quantitative analyses (Fig. 4I). We therefore concluded that a reduced level of apoptosis contributed to the densely packed RGC mosaics observed in the DT retina of early postnatal *Lhx2-Cre:Rptor^f/f^* mice.

### Domain-specific reduction in mTORC1-signalling leads to aberrant retinal morphology and RGC mosaics in adult mice

Next we examined whether the domain-specific decreases in mTORC1-signalling within the DT and VN retina altered RGC apoptosis at later developmental periods (Fig. 4E–H), as Raptor deficiency leads to continuous programmed cell death during nervous system development (Cloetta et al., 2013). Both the DT and VN regions in control animals at P21 exhibited solitary Casp3^+^ cells that were intermingled amongst evenly dispersed Brn3^+^ nuclei (Fig. 4E,F, yellow arrowheads). In contrast, an apparent increase in the number of apoptotic cells was observed in both domains within mutant animals (Fig. 4G,H), which was confirmed by quantification analyses (Fig. 4I).

**Fig. 5.**
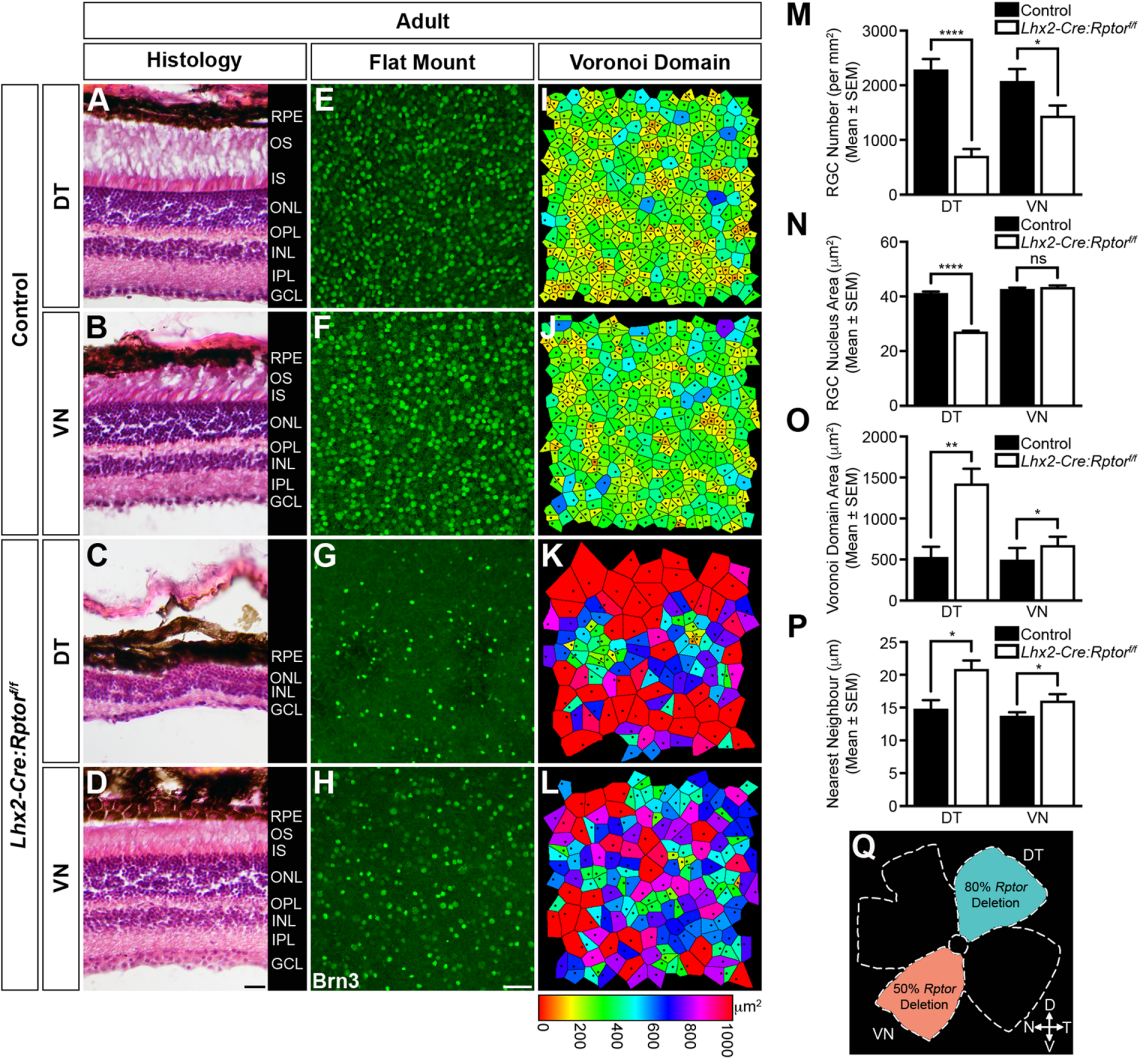
**Domain-specific reduction in mTORC1-signalling leads to aberrant retinal morphology and disorganised RGC mosaics in adult mice.** Enucleated eyes were harvested from control (*n*=8) and *Lhx2-Cre:Rptor^f/f^* mice (*n*=8) at 7 weeks of age and the morphological appearance of the retina in addition to the mosaic arrangement of RGCs in the DT and VN regions (Q) was determined. (A–D) Representative coronal eye sections taken from control mice (A,B) demonstrates that the retina in both the DT and VN regions exhibits a characteristic laminated structure with three nuclear layers (ONL, INL and GCL) being interconnected by two plexiform layers (OPL and IPL). In contrast, while the morphology of the retina in *Lhx2-Cre:Rptor^f/f^* mice (C,D) also displays comparable stratification, the apicobasal thickness of the DT region is reduced due to a decrease in the number of cells within the nuclear layers (C) while both regions exhibit a disorganised GCL. (E–H) Representative flat-mount images of control retinae demonstrate that the RGCs in both the DT (E) and VN (F) quadrants are dispersed in well-ordered arrays. In contrast, the Brn3^+^ cells in the DT (G) and VN regions (H) of *Lhx2-Cre:Rptor^f/f^* animals appear fewer in number with asymmetric distribution. (I–L) The spatial properties of the RGC mosaics were determined by Voronoi domain and nearest neighbour analyses. Representative heat map diagrams reveal greater variability in Voronoi domain areas and nearest neighbour distances within *Lhx2-Cre:Rptor^f/f^* animals (K,L) compared to control littermates (I,J). (M) Quantification of RGC number in the DT and VN quadrants of control and *Lhx2-Cre:Rptor^f/f^* mice. Mutant mice have a significant decrease in the mean number of Brn3^+^ cells in both domains. (N) Quantification of RGC nucleus area in the DT and VN quadrants of control and *Lhx2-Cre:Rptor^f/f^* mice. Mutant mice have a significant decrease in nucleus area in the DT region. (O,P) Quantitative analyses of the Voronoi domain (O) and nearest neighbour distances (P) demonstrate that RGCs within the DT and VN regions of *Lhx2-Cre:Rptor^f/f^* mice exhibit significantly larger domain areas and nearest neighbour distances compared to control littermates. The corresponding Voronoi domain and nearest neighbour distance histogram plots are presented in Fig. S12. (Q) Schematic diagram detailing the retinal quadrants imaged for the Voronoi domain and nearest neighbour analyses. All data represents the mean±s.e.m. Statistical differences were calculated using unpaired two-tailed Student's *t*-tests. *P*-values are denoted as follows: *P≤0.05, **P≤0.01 and ****P≤0.0001. Scale bar: (A–D) 100 µm; (E–H) 50 µm. Abbreviations: D, dorsal; DT, dorsotemporal; GCL, ganglion cell layer; INL, inner nuclear layer; IPL, inner plexiform layer; IS, inner segments; N, nasal; ONL, outer nuclear layer; OPL, outer plexiform layer; OS, outer segments; RPE, retinal pigment epithelium; T, temporal; V, ventral; VN, ventronasal.

This unexpected increase in RGC apoptosis within older postnatal *Lhx2-Cre:Rptor^f/f^* mice therefore provided an opportunity to investigate how sustained programmed cell death influenced both retinal morphology and RGC mosaics within adult animals (Fig. 5). Accordingly, enucleated eyes were harvested from mice at 7 weeks of age and the morphological appearance of the retinae was determined by histological staining. Coronal eye sections demonstrated that the DT and VN regions in control animals exhibited a characteristic laminated structure with the three nuclear layers (ONL, INL and GCL) being interconnected by two plexiform layers (Fig. 5A,B). In contrast, while the morphology of the retinae in *Lhx2-Cre:Rptor^f/f^* mice also displayed similar stratification, the apicobasal thickness of the DT region was greatly reduced (Fig. 5C) while both the DT and VN retina exhibited a disorganised GCL (Fig. 5C,D) which was populated by *ROSA26R^+^* cells (Fig. S11).

Given the aberrant morphology of the GCL in mutant animals we next examined the tangential arrangement of RGCs by flat-mount analyses (Fig. 5E–Q). In control retinae, a comparable number of Brn3^+^ cells were observed in both DT and VN quadrants (Fig. 5E,F). In contrast, a reduction in both Brn3^+^ cell number and nuclear area was observed in the DT retina of *Lhx2-Cre:Rptor^f/f^* mice (Fig. 5G) while only RGC number seemed to be affected within the VN domain (Fig. 5H) and this was subsequently confirmed by quantitative analyses (Fig. 5M,N). Moreover, the decrease in RGC numbers within the DT and VN domains of *Lhx2-Cre:Rptor^f/f^* animals appeared to disrupt the regularity of their mosaics. The spatial properties of these arrays were therefore determined by Voronoi domain and nearest neighbour approaches (Galli-Resta et al., 1997; Reese and Keeley, 2015). Both regions in wild-type animals (Fig. 5I,J) exhibited comparable spatial properties (Fig. 5O,P). However, the decreased number of RGCs in the DT (Fig. 5K) and VN regions (Fig. 5L) of *Lhx2-Cre:Rptor^f/f^* mice resulted in increased Voronoi domain areas and nearest neighbour distances (Fig. 5O,P) with the accompanying decrease in both VDRI and NNRI confirming a complete loss of mosaic regularity in the DT domain in particular (Fig. S12). Interestingly, this mosaic disarray phenotype was specific to *Lhx2-Cre:Rptor^f/f^* mice since the retina of heterozygous animals exhibited normal RGC tangential positioning despite the fact that the *Rptor* allele underwent recombination efficiencies similar to that observed within the VN domain of mutant mice (Figs S13 and S14).

### Aberrant RGC mosaics initiate retinogeniculate topography deficits in adult mice

**Fig. 6.**
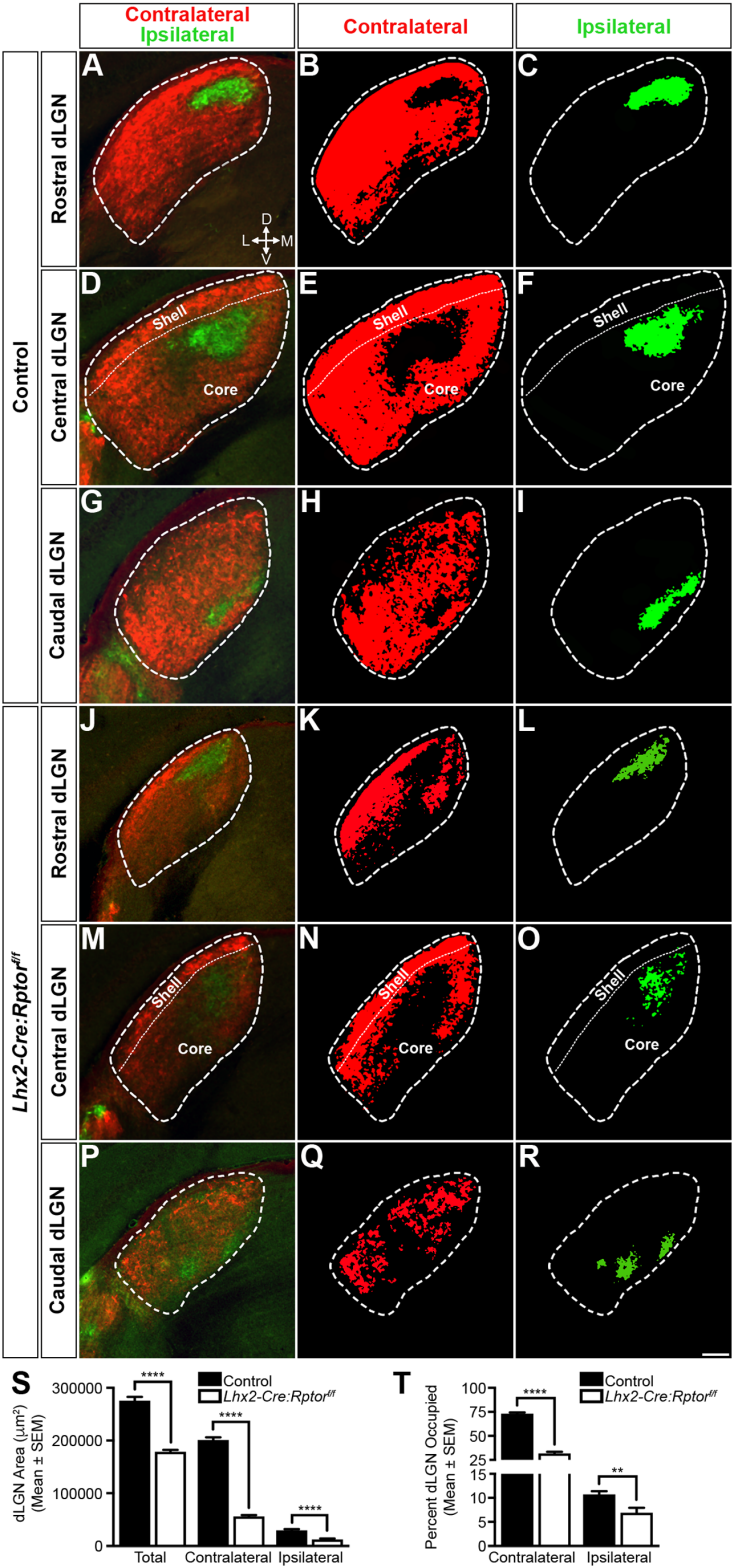
**Aberrant RGC mosaics induce loss of retinogeniculate termination topographies in adult mice.** Binocular projection patterns in the dLGN of control (*n*=8) and *Lhx2-Cre:Rptor^f/f^* (*n*=8) mice were visualised by intraocular injections of fluorescently labelled CTB at 6 weeks of age. (A–I) Representative coronal sections through the dLGN of control animals to visualise the termination topographies in rostral (A–C), central (D–F) and caudal (G–I) positions. Binocular projections (A,D,G) were well segregated into distinct contralateral (B,E,H) and ipsilateral inputs (C,F,I). (J–R) Representative coronal sections through the dLGN of mutant animals to show the retinogeniculate topographies in the rostral (J–L), central (M–O) and caudal (P–R) positions. The total dLGN area was reduced (J,M,P) and large unoccupied contralateral territories (K,N,Q) were observed. In addition, the termination area of the ipsilateral projection was also reduced in size and presented as clusters of distinct arbors (L,O,R). The contralateral and ipsilateral panels are presented as binarised images to allow for better visualisation of the termination topographies. Dashed lines define the border of the dLGN in all images. Dotted lines define the division between the shell and core domains in the central dLGN images (S). Quantitative area analysis demonstrated that the total dLGN and contributing contralateral and ipsilateral termination areas were significantly reduced in *Lhx2-Cre:Rptor^f/f^* mice when compared to control animals. (T) Quantitative occupation analysis demonstrated that the percentage of dLGN area occupied by the contralateral and ipsilateral projections was significantly reduced in *Lhx2-Cre:Rptor^f/f^* mice. All data represents the mean±s.e.m. Statistical differences were determined using unpaired two-tailed Student's *t*-tests. *P*-values are denoted as follows: **P≤0.01 and ****P≤0.0001. Scale bar: 100 µm. Abbreviations: D, dorsal; dLGN, dorsal lateral geniculate nucleus; L, lateral; M, medial; T, temporal; V, ventral.

Intraocular anterograde labelling was next performed on control and *Lhx2-Cre:Rptor^f/f^* mice at 6 weeks of age to determine whether the aberrant DT and VN RGC mosaics observed in mutant mice influenced their axonal termination topography within the retinorecipient dLGN (Fig. 6). Binocular inputs in control mice were segregated into a stereotypical pattern (Monavarfeshani et al., 2017) with a single ipsilateral patch being surrounded by the contralateral input throughout the whole rostrocaudal axis (Fig. 6A–I). In contrast, the area of dLGN in mutant animals (Fig. 6J–R) appeared smaller throughout the full extent of the nucleus (Fig. 6J,M,P) and the aberrant RGC mosaics influenced retinogeniculate topography. Uninnervated contralateral domains were observed (Fig. 6K,N,Q) with the loss of these axonal terminations in both rostral (Fig. 6K) and caudal (Fig. 6Q) positions correlating with the aberrant RGC mosaics present within the VN and DT retina, respectively (Cang and Feldheim, 2013). Interestingly, axonal input was consistently maintained within the dorsolateral shell of the dLGN in *Lhx2-Cre:Rptor^f/f^* animals with the majority of the unoccupied contralateral domains residing in the ventromedial core of the nucleus (Fig. 6N). Moreover, the topography and position of the ipsilateral projection was skewed in mutant mice (Fig. 6L,O,R) with ectopic arbors being observed (Fig. S15). These regions of aberrant innervation were primarily detected within central (Fig. 6O) and caudal (Fig. 6R) positions of the dLGN. The severity of retinogeniculate topography disruption was subsequently confirmed by quantitative analyses. Total dLGN and contributing contralateral and ipsilateral area were significantly reduced in *Lhx2-Cre:Rptor^f/f^* mice (Fig. 6S). The percentage area occupied by each input was therefore quantified to accommodate for these total area differences. Accordingly, the percentage area occupied by both contralateral and ipsilateral projections were significantly reduced in *Lhx2-Cre:Rptor^f/f^* mice (Fig. 6T).

### The cumulative effects of *Rptor*-ablation leads to visually mediated behavioural deficits

**Fig. 7.**
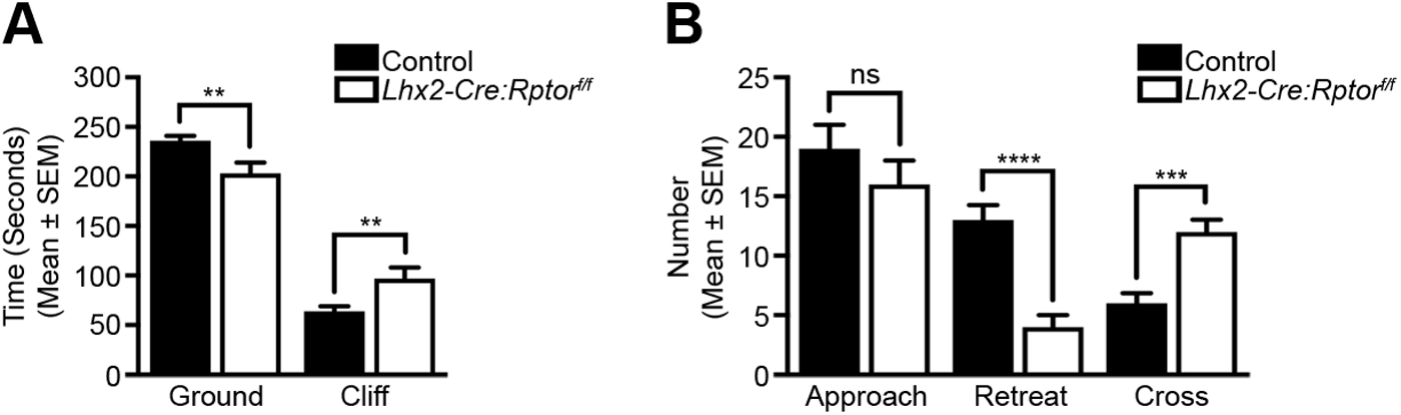
**The cumulative effects of *Rptor*-ablation lead to visual behaviour deficits.** Visual cliff analyses of control (*n*=20) and *Lhx2-Cre:Rptor^f/f^* (*n*=13) mice between 6 and 10 weeks of age. (A) Visual cliff data analysed for total time spent on either the ground or cliff side of the testing arena. *Lhx2-Cre:Rptor^f/f^* mice spend significantly less time on the ground and consequently more time on the cliff side. (B) Visual cliff data analysed for total number of (i) approaches to the central beam, (ii) retreats to the ground side following an approach and (iii) crosses to the cliff side following an approach. Both groups made a comparable number of approaches towards the central beam but *Lhx2-Cre:Rptor^f/f^* mice made significantly more crosses onto the cliff side of the testing arena with a consequent reduction in the number of retreats. All data represents the mean±s.e.m. Statistical differences were calculated using unpaired two-tailed Student's *t*-tests. *P*-values are denoted as follows: **P≤0.01, ***P≤0.001 and ****P≤0.0001.

Control and mutant animals were finally subjected to visual cliff testing (Fox, 1965) to determine whether the aberrant RGC mosaics and associated loss of dLGN inputs would lead to visually mediated behavioural deficits (Fig. 7). To begin these analyses all mice were placed on a central beam that divided an open field arena into two areas designated the ground and cliff sides and their behaviour was monitored. In a majority of instances control mice approached the central beam and inspected the cliff before retreating to the ground region of the testing arena (Movie 1). In contrast, *Lhx2-Cre:Rptor^f/f^* mice frequently crossed over the dividing beam and onto the cliff side without hesitation (Movie 2). Accordingly, mutant mice spent significantly more time on the cliff side (Fig. 7A) which suggested that *Lhx2-Cre:Rptor^f/f^* animals do not exhibit a preference for the ground side of the testing arena. In addition, we also observed that although both groups made a comparable number of exploratory approaches towards the central dividing beam, the mutant mice made significantly more crosses onto the cliff side of the testing arena (Fig. 7B). In combination these results demonstrated that the aberrant RGC mosaics and associated loss of retinogeniculate topography observed in *Lhx2-Cre:Rptor^f/f^* mice induced visually mediated behaviour deficits

## DISCUSSION

This manuscript identifies mTORC1 as a critical regulator of various aspects of visual pathway development and function. *Lhx2-Cre* mediated *Rptor* deletion within RPCs led to (i) aberrant RGC neurogenesis, (ii) abnormal RGC mosaics, (iii) retinogeniculate topography defects and (iv) associated visual deficits. This study therefore contributes to the current knowledge regarding the role of mTORC1-signalling during the development of diverse organs and tissues (Bentzinger et al., 2008; Cloetta et al., 2013; Daikoku et al., 2013; Fitter et al., 2017; Ni et al., 2017).

Deletion of *Rptor* in RPCs led to changes in cell cycle progression that resulted in a reduction of total retina size that was already discernible within embryonic *Lhx2-Cre:Rptor^f/f^* mice. These observations agree with previous reports where either *mTOR* or *Rptor*-ablation led to proliferation deficits and tissue volume decreases within various CNS domains (Cloetta et al., 2013; Ka et al., 2014). Our data is therefore consistent with the evolutionary conserved role of mTORC1 during cell cycle progression (Fingar and Blenis, 2004). Interestingly, a recent publication demonstrated that *Chx10-Cre* mediated *Rptor*-ablation in RPCs similarly led to an inhibition of cell cycle progression during retinal development (Choi et al., 2018). Such deficits induced extensive apoptosis of Raptor-null cells during embryonic development and led to the production of a smaller retina in postnatal animals that was composed predominantly of wild-type neurons (Choi et al., 2018). While we also noted similar proliferation deficits upon *Rptor* ablation, the selective removal of mutant cells was not observed. Instead, the adult retina of *Lhx2-Cre:Rptor^f/f^* mice was populated with Raptor-deficient RGCs particularly within the DT region. Differences in the temporal and spatial kinetics of *LoxP* recombination mediated by the *Chx10-Cre* transgene must therefore underlie the contrasting phenotypic outcomes observed following *Rptor*-ablation in this recent study (Choi et al., 2018; Rowan and Cepko, 2004).

An over-production of RGCs was observed in embryonic *Lhx2-Cre:Rptor^f/f^* mice with the greatest number of cells being observed within the DT retina which itself exhibited a complete absence of mTORC1-signalling. Such observations suggest that mTORC1 mediates control over cell-fate decisions and corroborates previous studies in both *Drosophila* and mouse (Bateman and McNeill, 2004; Jones et al., 2015). These authors demonstrated that precocious mTORC1 activation led to an over-production of distinct neuronal classes within the developing eye. Taken together, we can conclude that balanced mTORC1 activity is required to coordinate the timing of cell-fate decisions and that any anomalous fluctuations leads to aberrant neurogenesis. How mTORC1 influences the behaviour of temporal identity factors therefore provide intriguing avenues for future investigation since these molecules are involved in regulating the ordered progression of RPC differentiation during mouse eye development (Mattar and Cayouette, 2015). Of particular interest to our study would be the effects of *Rptor*-ablation upon the temporal expression of Castor since conditional deletion of *CasZ1* within mouse RPCs leads to an over-production of early born neuronal subtypes including RGCs (Mattar et al., 2015).

*Rptor*-deletion influenced the mosaic arrangement of RGCs and highlights that this pathway mediates integral cell positioning roles during early postnatal development of the mouse retina. The reduced level of apoptosis detected in the DT retina of *Lhx2-Cre:Rptor^f/f^* animals was presumably the primary driving mechanism behind this compact mosaic arrangement. This was reminiscent of *Bax*-null and *Bcl2* transgenic mice where defects in programmed cell death also lead to increased numbers of RGCs and their consequent aberrant tangential distribution (Keeley et al., 2012; Strettoi and Volpini, 2002). Taken together, it can be concluded that one consequence of reduced RGC apoptosis during postnatal retinal development is the loss of tangential symmetry. Future studies will therefore be aimed at assessing the mosaic distribution of other neuronal classes within the DT and VN retina of mutant animals. Of particular interest to this current study are starburst amacrine cell mosaics since these cholinergic neurons are critical for RGC function in addition to being the source of stage II nAChRβ2-driven retinal waves that are critical for dLGN axonal refinement (Feller et al., 1997; Stacy and Wong, 2003).

Notable differences were observed in the retinogeniculate topography within *Lhx2-Cre:Rptor^f/f^* mice. Firstly, elevated apoptosis and consequent RGC mosaic disarray observed within the VN and DT retina of adult mice led to the production of large unoccupied contralateral territories within the rostral and caudal dLGN, respectively (Cang and Feldheim, 2013). Interestingly, the majority of these unoccupied domains were always observed within the ventromedial core of the dLGN and suggests the possibility that RGC subtypes that project to this deeper sublamina are more susceptible to *Rptor* deletion in comparison to those classes that project to the superficial shell region (Huberman et al., 2009; Kim et al., 2010). Another difference was that the topography of the ipsilateral projection within *Lhx2-Cre:Rptor^f/f^* mice was consistently altered and ectopic termination arbors were routinely positioned throughout the central and caudal dLGN. This aberrant topography was reminiscent to that previously reported for mouse models where precocious mTORC1-signalling led to disrupted dLGN termination patterns and illustrates that this pathway is critical for development of the retinogeniculate projection (Jones et al., 2015; Nie et al., 2010).

Several possible mechanisms could account for the ectopic ipsilateral phenotype observed in *Lhx2-Cre:Rptor^f/f^* mice. A previous publication reported that mTORC1 signalling cooperated with the ephrin-Eph receptor system to control projection topography within the dLGN (Nie et al., 2010). Similar axon guidance defects could therefore be operating within *Lhx2-Cre:Rptor^f/f^* mice and function under RGC-autonomous mechanisms since the *Lhx2-Cre* transgene is not expressed within the dLGN (Jones et al., 2015). Alternatively, *Lhx2-Cre:Rptor^f/f^* mice might have axonal refinement defects within the dLGN as this process is driven by coordinated waves of neural activity that spread across the entire surface of the retina prior to the onset of vision (Feller et al., 1997). Of particular interest to this study are stage II nAChRβ2-driven waves that occur during the first week of postnatal development and overlap with the RGC mosaic formation and associated dLGN projection refinement (Bansal et al., 2000; Reese and Keeley, 2015). Moreover, that *nAChRβ2*-null mice exhibit ipsilateral retinogeniculate topography deficits that phenocopy those observed in *Lhx2-Cre:Rptor^f/f^* animals (Cang et al., 2005; Grubb et al., 2003; Rossi et al., 2001; Young, 1984) highlights the possibility that aberrant retinal activity during postnatal RGC mosaic formation might also contribute to the dLGN topography documented in this study. However, we also cannot exclude the possibility that the surviving RGCs undergo adaptive reorganisation and sprout ectopic axon terminals as a direct response to the elevated apoptosis observed in adult *Lhx2-Cre:Rptor^f/f^* mice.

Whatever the mechanism, the observed aberrant retinogeniculate termination patterns profoundly influenced visually mediated behaviour in *Lhx2-Cre:Rptor^f/f^* mice. This observation corroborates previous studies where RGC projection mismatch led to deficits when assessing the functionality of the ventral visual field (de Lima et al., 2012; Leamey et al., 2007). Taken together, these studies demonstrate the importance of the DT retina and retinogeniculate pathway for stereoscopic depth perception within binocular animals (Seabrook et al., 2017). However, we concede that the aberrant lamination morphology within the DT retina of adult *Lhx2-Cre:Rptor^f/f^* mice also presumably contributes to the impaired visual behaviour observed in this study. Future studies will therefore be aimed at assessing the cohesiveness of the visual field sampled by the VN retina and to correlate this functionality to the severity of the aberrant RGC mosaics observed within this region relative to that observed in the DT retina of *Lhx2-Cre:Rptor^f/f^* animals.

All previous publications involving the *Lhx2-Cre* transgene resulted in target gene recombination patterns that mirrored that of the *ROSA26R* reporter allele (Hagglund et al., 2013, 2011, 2017; Jones et al., 2015). In contrast, a distinct pattern of *Rptor*-ablation was reproducibly generated in the retina of *Lhx2-Cre:Rptor^f/f^* mice where the DT region consistently exhibited the greatest level of allelic recombination. Moreover, this pattern of incomplete *Rptor* deletion did not correlate with the global ablation pattern of the *ROSA26R* reporter allele. Such an observation therefore demonstrates the importance of defining the extent of target gene deletion in place of relying on the spatial activity of a surrogate reporter to predict Cre-mediated recombination patterns (Kinare et al., 2019). The mechanism underlying the selective resistance of the *Rptor* allele to *Lhx2-Cre*-mediated recombination remain unclear but it occurred during a developmental window (E8.25 to E12.5) that coincided with optic vesicle to optic cup transition (Graw, 2010; Hagglund et al., 2011). It can therefore be concluded that the progenitor pool that forms the DT retina (i) originates from a specific cohort of neighbouring cells within the optic pit and (ii) is more susceptible to Cre-mediated recombination of the *Rptor* allele compared to the remaining eye-committed progenitor cells. Moreover, our current observations confirm that the various developmental processes that underlie the formation of the rudimentary eye structure are independent of mTORC1 function (Jones et al., 2015).

In conclusion, this manuscript establishes a critical cell-autonomous role for mTORC1 signalling during RGC development and function. Such observations establish a foundation for future studies concerning the developmental roles of mTORC1 within other retinal neurons. Such systematic approaches will help build a unified understanding into how mTORC1 contributes to the establishment of a functional visual pathway in vertebrate species. Furthermore, that RGC mosaics also function as a guidance scaffold for retinal vascularisation (O'Sullivan et al., 2017) highlights that *Lhx2-Cre:Rptor^f/f^* mice are also an ideal model to investigate cell non-autonomous mechanisms driven by mTORC1-signalling during eye development.

## MATERIALS AND METHODS

### Animals

All animal experiments were approved by the Animal Review Board at the Court of Appeal of Northern Norrland (A49-13 and A36-2018). The derivation and genotyping of *Tg(Lhx2-Cre)1Lcar* transgenic mice (written as *Lhx2-Cre*) (Hagglund et al., 2011), *Rptor^tm1.1Dmsa^* floxed mice (written as *Rptor^+/f^* or *Rptor^f/f^*) (Sengupta et al., 2010) and *Gt(ROSA)26Sor^tm1sor^* reporter mice (written as *ROSA26R*) (Soriano, 1999) have been previously described. The genotype of all animals was determined by PCR analysis of genomic DNA extracted from ear punch biopsies (Sambrook and Russell, 2001). Breeding *Lhx2-Cre:Rptor^+/f^* and *Rptor^f/f^* mice or *Lhx2-Cre:Rptor^+/f^* and *Rptor^f/f^*:*ROSA26R* mice generated all experimental animals. The morning of the vaginal plug was considered as E0.5. Littermates lacking the *Lhx2-Cre* transgene were used as controls for all experiments apart from the lineage tracing analyses where *Lhx2-Cre:Rptor^+/f^*:*ROSA26R* mice were employed as controls. All analyses were carried out on *129/Sv:CBA:C57BL/6* and *129/Sv:CBA:C57BL/6:NMRI* mixed genetic backgrounds. No phenotypic penetrance variability was observed between the different strains. Both males and females were used for experimental analyses.

### Histology

Enucleated eyes were fixed in 2% (w/v) glutaraldehyde in PBS overnight at 4°C. Eyes were immersed in 70% (v/v) ethanol and paraffin embedded. Paraffin sections (10 µm) were cleared by 2×5 min incubation in xylenes before rehydration though a graded series of ethanol [99.5%, 95%, 90% and 80% (v/v) in PBS]. Haematoxylin and Eosin staining was performed as previously described (Hagglund et al., 2011).

### Immunoblotting

The retina was removed from enucleated eyes and snap frozen in liquid nitrogen. Soluble protein extraction and immunoblotting was performed as previously described (Hagglund et al., 2017). The following primary antibodies and dilutions were used: GAPDH (1:30,000, Cell Signaling Technology, #2118), Raptor (1:1000, Cell Signaling Technology, #2280), S6 (1:2000, Cell Signaling Technology, #2217), pS6^S235/236^ (1:1000, Cell Signaling Technology, #4857), pS6^S240/244^ (1:2000, Cell Signaling Technology, #5364).

### Quantitative PCR

A heated needle was used to burn an orientation mark at the dorsal pole of the eye prior to enucleation. The cornea and lens were removed and a radial incision was made from the orientation mark along the dorsal axis towards the optic disc. The pigment epithelium, iris and ciliary body were then peeled away and the retina was further dissected by radial incisions along the temporal, ventral and nasal axes to yield four equal tissue quadrants. Genomic DNA was extracted from these quadrants (Sambrook and Russell, 2001) and used as the template for qPCR using the FastStart Universal SYBR Green Master (Rox) (Roche) and CFX Connect™ Real-Time System (Bio-Rad). All reactions were performed in duplicate using the following oligonucleotides designed against exon 4 (R4F 5′-GAGCCTCGACCCTACTGTGG-3′ and R4R 5′-GGCGCAGAGATGTGCAAAGT-3′) and exon 6 (R6F 5′-CATCCAGTTGGCAGCGTGTG-3′ and R6R 5′-GGAGGTCAGGGATCATGGGC-3′) of the mouse *Rptor* gene (NCBI 74370).

### Lineage tracing

For cryosection analyses, heads or enucleated eyes were fixed in 4% (w/v) PFA in PBS for 30 min on ice. The tissues were then equilibrated overnight at 4°C in 30% (w/v) sucrose in PBS and embedded in OCT compound (Sakura Finetek). For flat-mount analyses, a heated needle was first used to burn an orientation mark at the dorsal pole of the eye prior to enucleation. Isolated eyes were then placed in separate wells of a 48-well plate and fixed in 4% (w/v) PFA in PBS for 10 min on ice. Retinae were then dissected out and four incomplete radial incisions were made along the dorsal, temporal, ventral and nasal axes to yield four petals attached to one another at the central optic disc region. A separate incision was also placed in the dorsonasal petal for orientation purposes. The retinae were then fixed for a further 30 min in 4% (w/v) PFA in PBS on ice. Lineage tracing analyses were performed on cryosections (10 µm) or flat-mount retina as described previously (Hagglund et al., 2011).

### Immunohistochemistry

For cryosection analyses, heads or enucleated eyes were fixed in 4% (w/v) PFA in PBS for up to 2 h on ice. The tissues were equilibrated overnight at 4°C in 30% (w/v) sucrose in PBS and embedded in OCT compound (Sakura Finetek). Flat-mount dissections were done as described above for lineage tracing. Immunohistochemistry was performed on cryosections (10 µm) or flat-mount retina as previously described (Cantrup et al., 2012; Ma et al., 2007). An additional blocking step involving M.O.M Blocking Reagent (Vector Labs) was used in all experiments involving monoclonal primary antibodies. The following primary antibodies and dilutions were used: BrdU^Alexa555^ (1:20, BD Pharmingen, #560210); Brn3 (1:50, SCBT, #sc6026); Casp3 (1:500, Abcam, #ab13847); NEFH, (1:1000, Covance, #SMI31R); PH3 (1:100, Millipore, #06-570); pS6^S235/236^ (1:200, Cell Signaling Technology, #4857); pS6^S240/244^ (1:200, Cell Signaling Technology, #5364). All immunohistochemistry antibodies have been independently verified in our previous studies (Hagglund et al., 2017; Jones et al., 2015).

### Proliferation analyses

Pregnant dams were intraperitoneally injected with BrdU (50 mg/kg body weight). The mice were euthanised 4 h later and the embryos collected. Embryonic heads were fixed in 4% (w/v) PFA in PBS for 2 h on ice and then equilibrated overnight at 4°C in 30% (w/v) sucrose in PBS prior to being embedded in OCT compound (Sakura Finetek). Cryosections (10 µm) were washed in PBS and then denatured in 10 mM sodium citrate buffer (pH 6) for 10 min at 95°C. The antigen retrieved slides were then allowed to cool to room temperature before being washed again in PBS. Immunohistochemistry was performed as described above.

### *In situ* hybridisation

Embryonic heads were fixed in 4% (w/v) PFA in PBS for 2 h on ice and equilibrated in 30% (w/v) sucrose in PBS overnight at 4°C. Tissues were then embedded in OCT compound (Sakura Finetek). *In situ* hybridization on cryosections (10 µm) was performed as previously described (Schaeren-Wiemers and Gerfin-Moser, 1993). The following IMAGE clone (Source Bioscience) was used for riboprobe generation: *Math5* (Clone 6824509).

### Intraocular anterograde labelling

Mice were anesthetised by an intraperitoneal injection of Hypnorm (0.079 mg/ml fentanyl citrate and 2.5 mg/ml fluanisone) and Midazolam (1.25 mg/ml) at 10 µl/g. Fluorescently labelled cholera toxin subunit-B (CTB) was diluted in PBS (1 mg/ml) and anesthetised mice subsequently received intraocular injections of CTB^Alexa488^ (left eye) and CTB^Alexa594^ (right eye) (3 µl per eye). Injections were performed using pulled glass needles coupled to Hamilton syringes. Operated mice were returned to their home cage and allowed to recover on heated pads. The mice were transcardially perfused 72 h later with 4% (w/v) PFA in PBS. The brain was removed and further fixed overnight at 4°C in 4% (w/v) PFA in PBS prior to being embedded in 4% (w/v) agarose in PBS. Coronal vibratome sections (100 µm) were counterstained with DAPI and mounted with Aqua Polymount (Polysciences Inc.).

### Visual cliff test

Mice were anesthetised by an intraperitoneal injection of Hypnorm (0.079 mg/ml fentanyl citrate and 2.5 mg/ml fluanisone) and Midazolam (1.25 mg/ml) at 10 µl/g. Once sedated their vibrissae were removed by an electric hair trimmer. Mice were subsequently returned to their home cage and allowed to recover on a heated pad and subjected to behavioural testing 24 h later. An open field apparatus (60×60×20-cm) comprising of white walls and a clear acrylic base was used as the testing arena. A high-contrast checkerboard pattern was attached to the underside of one half of the base and white wooden beam (60×5×1-cm) was placed inside the apparatus so as to divide the testing arena into two halves designated the ground (high-contrast floor) and cliff (clear floor) side. The apparatus was then placed such that the cliff protruded from the laboratory bench revealing a drop of approximately 80 cm. To begin the test the mice were placed on the central beam and their behaviour was monitored for 5 min using a digital video camera suspended above the testing arena. An approach was defined as moving from the ground side towards the cliff and placing both front limbs on the central beam. Retreating was defined as contacting the ground side of the testing arena with all four limbs following an approach. Crossing was defined as the animal completely passing over the midline beam and placing all four limbs on the cliff side following an approach.

### Image analyses

Images were captured using either a LSM 710 confocal microscope (Zeiss) or an Eclipse E800 microscope (Nikon). Images were compiled and analysed using Fiji (Schindelin et al., 2012), CellProfiler (Carpenter et al., 2006; Kamentsky et al., 2011), Adobe Photoshop and Adobe Illustrator. Cell count quantifications were performed using customised pipelines written in CellProfiler. Voronoi domain, nearest neighbour and retinogeniculate topography analyses were performed using customised macros written in Fiji. All imaging scripts used in this manuscript are available from the corresponding author upon request.

### Statistical analyses

Statistical analyses were performed using Prism7 (GraphPad Software). Quantification analyses were performed blind to genotype. Unpaired two-tailed Student's *t*-tests were used to determine statistical significances. Error bars in all figures represent the standard error of the mean (s.e.m.). The number of animals analysed (*n*) for each statistical test are given in the respective figure legend. *P*-values are indicated as follows: *P≤0.05, **P≤0.01, ***P≤0.001, ****P≤0.0001.

## Supplementary Material

Supplementary information
