# Supplementary material for: Reduced mTORC1-signalling in retinal progenitor cells leads to visual pathway dysfunction
Source: Biol Open. 2019 Jul 8;8(8):bio044370. doi: 10.1242/bio.044370 (PMC6737973; doi:10.1242/bio.044370)
Supplement: Supplementary information [file biolopen-8-044370-s1.pdf]

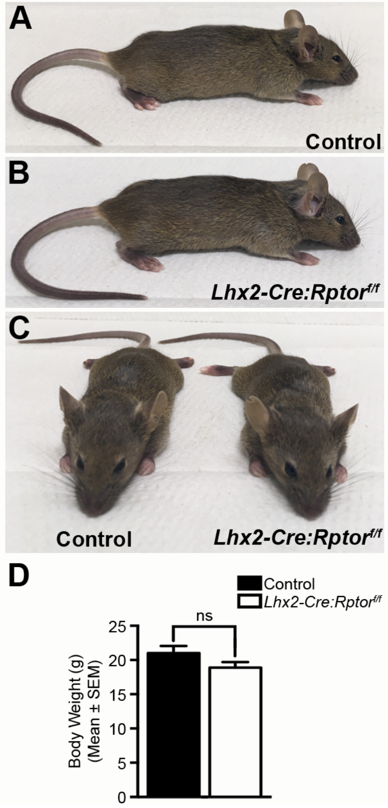

Figure S1. Control and *Lhx2-Cre:Rptor$^{f/f}$* mice are physically indistinguishable from each other. (A – C) Physical appearance of control and *Lhx2-Cre:Rptor$^{f/f}$* mice. (D) Control (n = 7) and *Lhx2-Cre:Rptor$^{f/f}$* mice (n = 8) exhibit comparable body weights. All mice were imaged and weighed at 7 weeks of age. The data represents the mean ± SEM.

Biology Open • Supplementary information

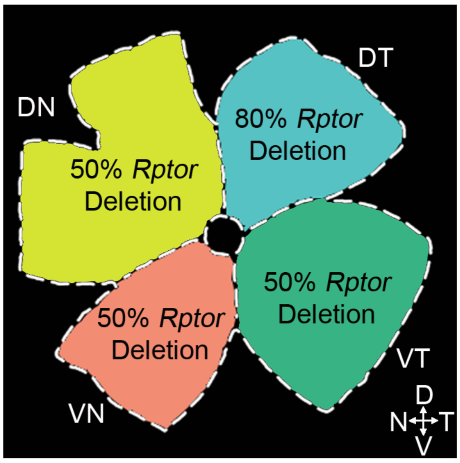

Figure S2. Schematic diagram illustrating the domain-specific pattern of *Rptor*-ablation in the retina of *Lhx2-Cre:Rptor*$^{f/f}$ mice. A characteristic pattern of *Rptor* recombination is observed within the retina of *Lhx2-Cre:Rptor*$^{f/f}$ mice with an approximate 80% deletion level being observed in the DT region while the remaining quadrants exhibit close to a 50% ablation rate. Abbreviations: D, dorsal; DN, dorsonasal; DT, dorsotemporal; N, nasal; T, temporal; V, ventral; VN, ventronasal; VT, ventrotemporal.

Biology Open • Supplementary information

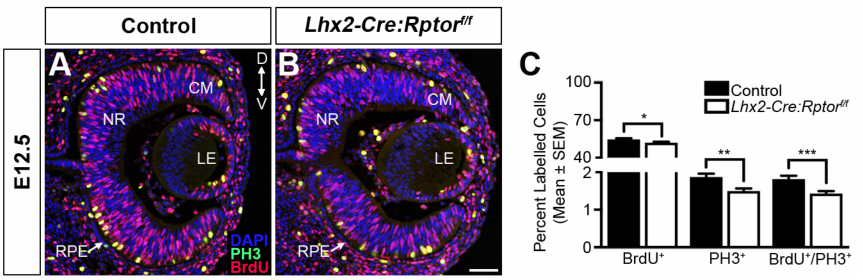

Figure S3. Conditional deletion of *Rptor* leads to reduced RPC proliferation. Embryos were harvested from control (*n* = 8) and *Lhx2-Cre:Rptor$^{f/f}$* (*n* = 8) mice and the number of BrdU^{+}, PH3^{+} and BrdU^{+}/PH3^{+} cells was assessed by immunohistochemistry. (A – B) Representative coronal sections through the retina of control (A) and *Lhx2-Cre:Rptor$^{f/f}$* (B) mice at E12.5 demonstrates an apparent reduction in the number of proliferating (BrdU^{+}) and mitotic (PH3^{+}) cells in mutant animals. (C) Quantitative analysis demonstrates that the percentage of BrdU^{+}, PH3^{+} and double labeled cells was significantly reduced in *Lhx2-Cre:Rptor$^{f/f}$* mice. All data represents the mean ± SEM. Statistical differences were calculated using unpaired two-tailed Student's t-tests. p values are denoted as follows: *$p \leq 0.05$, **$p \leq 0.01$ and ***$p \leq 0.001$. Scale bar: (A – B) 50 μm. Abbreviations: CM, ciliary margin; D, dorsal; LE, lens; NR, neural retina; RPE, retinal pigment epithelium; V, ventral.

Biology Open • Supplementary information

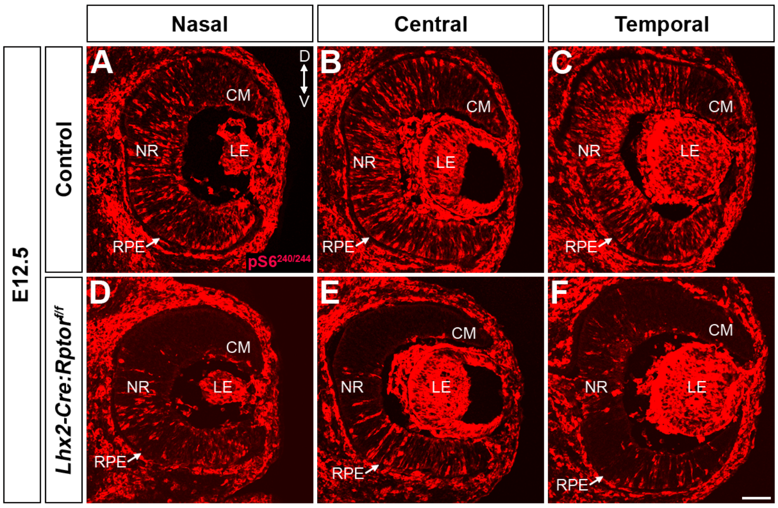

Figure S4. Reduced mTORC1-signaling leads to decreased pS6 levels at the commencement of RGC neurogenesis. mTORC1-activity was assessed by immunohistochemistry at the onset of RGC neurogenesis (E12.5) in control (n = 8) and *Lhx2-Cre:Rptor*$^{f/f}$ (n = 8) mice. (A – F) Representative coronal sections through the nasal to temporal retinal axis of control (A – C) and mutant (D – F) animals demonstrates reduced mTORC1-signaling in *Lhx2-Cre:Rptor*$^{f/f}$ mice. Scale bar: (A – F) 50 µm. Abbreviations: CM, ciliary margin; D, dorsal; LE, lens; NR, neural retina; RPE, retinal pigment epithelium; V, ventral.

Biology Open • Supplementary information

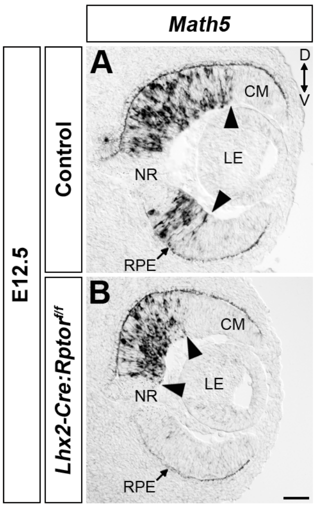

Figure S5. Reduced mTORC1-signaling leads to decreased *Math5* spatial expression. *In situ* hybridisation analyses of *Math5* expression in control (n = 4) and *Lhx2-Cre:Rptor*$^{f/f}$ (n = 4) mice at E12.5. (A – B) Representative coronal eye sections through the temporal retina of control (A) and mutant (B) animals demonstrates a reduced spatial expression domain for *Math5* in *Lhx2-Cre:Rptor*$^{f/f}$ mice. The black triangles represent the dorsal and ventral expression boundaries, respectively. Scale bar: (A – B), 50 µm. Abbreviations: CM, ciliary margin; D, dorsal; LE, lens; NR, neural retina; RPE, retinal pigment epithelium; V, ventral.

Biology Open • Supplementary information

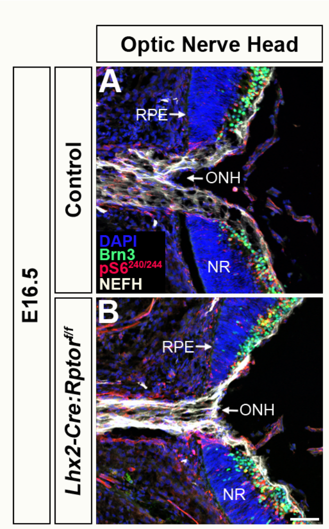

Figure S6. An increased number of RGC axonal projections exit the eye in *Lhx2-Cre:Rptor$^{f/f}$* mice. (A - B) Coronal sections of the central retina in control (*n* = 8) (A) and *Lhx2-Cre:Rptor$^{f/f}$* (*n* = 12) (B) mice at E16.5 demonstrates that an increased number of NEFH^{+} axons exit the eye in mutant animals. Scale bar: (A – B) 50 µm. Abbreviations: NR, neural retina; ONH, optic nerve head; RPE, retinal pigment epithelium.

Biology Open • Supplementary information

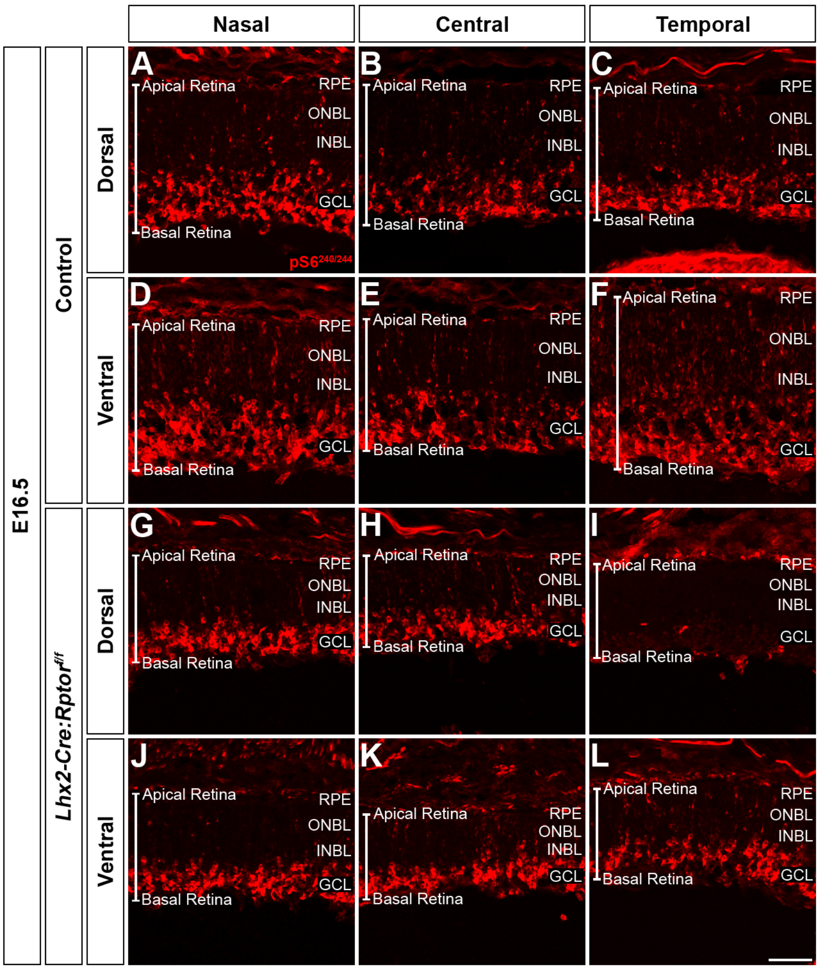

Figure S7. Reduced mTORC1-signaling leads to decreased pS6 levels during RGC radial migration. mTORC1-activity was assessed by immunohistochemistry during RGC radial migration (E16.5) in the retina of control (n = 8) and *Lhx2-Cre:Rptor$^{f/f}$* (n = 12) mice. (A – L) Representative coronal sections through the nasal to temporal axis of control (A – F) and *Lhx2-Cre:Rptor$^{f/f}$* (G – L) animals reveals a reduction in apicobasal thickness (brackets) and mTORC1-signaling throughout the whole extent of the nasal to temporal axis of *Lhx2-Cre:Rptor$^{f/f}$* animals with the DT region (I) exhibiting a complete lack of pS6$^{S240/244}$ immunoreactivity. Scale bar: (A – L) 50 µm. Abbreviations: GCL, ganglion cell layer; INBL, inner neuroblastic layer; ONBL, outer neuroblastic layer; RPE, retinal pigment epithelium.

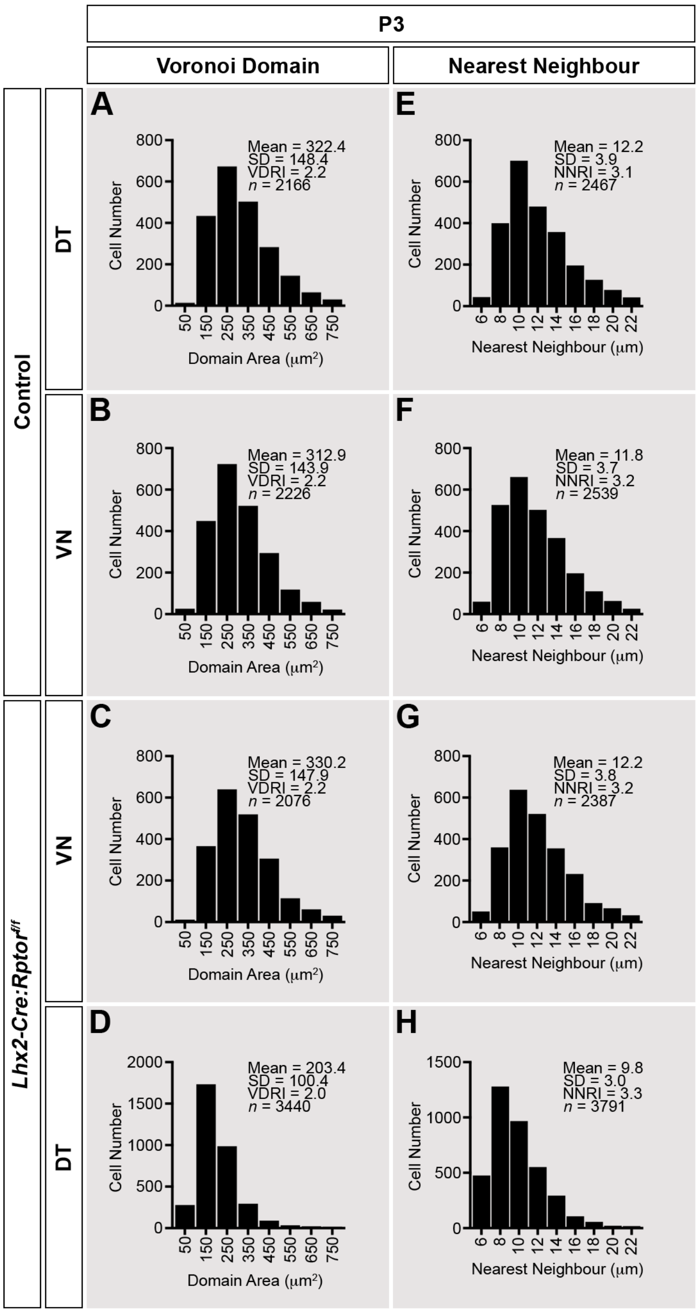

Figure S8. Histogram frequency plots of RGC mosaics at P3. (A – H) Retinae were harvested from control (n = 6) and *Lhx2-Cre:Rptor*$^{f/f}$ (n = 6) mice at P3 and the spatial properties of RGC mosaics in the DT and VN domains were determined. (A – D) Histogram frequency plots of Voronoi domain areas (μm^2) in control (A – B) and mutant (C – D) mice reveal comparable mean area values in addition to a normal distribution of cell territories in the DT and VN domains of wild type animals and in the VN region of *Rptor*-ablated mice (A – C). In contrast, a significantly reduced mean area value and a right-skewed distribution of Voronoi domain areas were present in the DT domain of *Lhx2-Cre:Rptor*$^{f/f}$ animals (D). (E – H) Histogram frequency plots of nearest neighbour distances (μm) in control (E – F) and

Biology Open • Supplementary information

mutant (G – H) mice reveal comparable mean distance values in addition to a normal distribution of RGC somata in the DT and VN domains of wild type animals and in the VN region of *Rptor*-ablated mice (E – G). In contrast, a significantly reduced mean distance value and a right-skewed distribution of nearest neighbour distance were present in the DT domain of *Lhx2-Cre:Rptor*$^{f/f}$ animals (H). The VDRI and NNRI were calculated for each domain and both control and *Lhx2-Cre:Rptor*$^{f/f}$ mice exhibited similar values. Abbreviations: DT, dorsotemporal; *n*, number; NNRI, nearest neighbour regularity index; P, postnatal; RGCs, retinal ganglion cells; SD, standard deviation; VN, ventronasal; VDRI, Voronoi domain regularity index.

Biology Open • Supplementary information

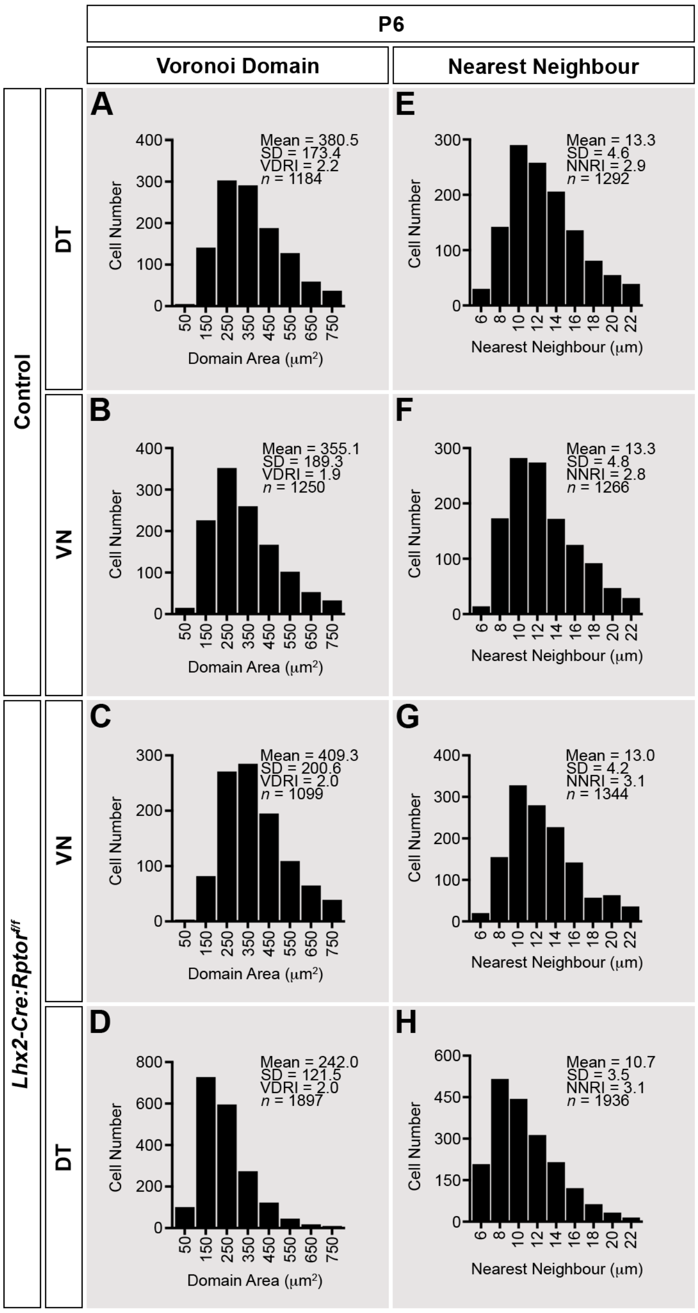

Figure S9. Histogram frequency plots of RGC mosaics at P6. (A – H) Retinae were harvested from control (n = 4) and *Lhx2-Cre:Rptor*$^{f/f}$ (n = 4) mice at P6 and the spatial properties of RGC mosaics in the DT and VN domains were determined. (A – D) Histogram frequency plots of Voronoi domain areas (μm^2) in control (A – B) and mutant (C – D) mice reveal comparable mean area values in addition to a normal distribution of cell territories in the DT and VN domains of wild type animals and in the VN region of *Rptor*-ablated mice (A – C). In contrast, a significantly reduced mean area value and a right-skewed distribution of Voronoi domain areas were present in the DT domain of *Lhx2-Cre:Rptor*$^{f/f}$ animals (D). (E – H) Histogram frequency plots of nearest neighbour distances (μm) in control (E – F) and

Biology Open • Supplementary information

mutant (G – H) mice reveal comparable mean distance values in addition to a normal distribution of RGC somata in the DT and VN domains of wild type animals and in the VN region of *Rptor*-ablated mice (E – G). In contrast, a significantly reduced mean distance value and a right-skewed distribution of nearest neighbour distance were present in the DT domain of *Lhx2-Cre:Rptor*$^{f/f}$ animals (H). The VDRI and NNRI were calculated for each domain and both control and *Lhx2-Cre:Rptor*$^{f/f}$ mice exhibited similar values. Abbreviations: DT, dorsotemporal; *n*, number; NNRI, nearest neighbour regularity index; P, postnatal; RGCs, retinal ganglion cells; SD, standard deviation; VN, ventronasal; VDRI, Voronoi domain regularity index.

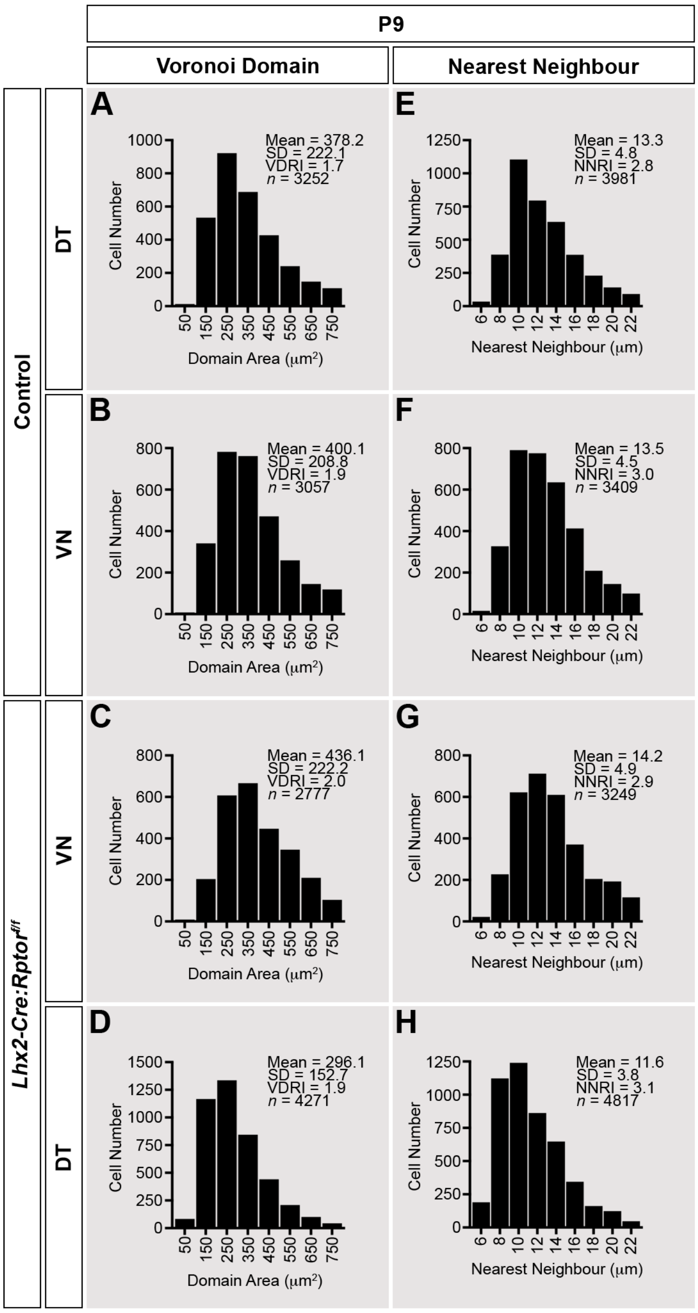

Figure S10. Histogram frequency plots of RGC mosaics at P9. (A – H) Retinae were harvested from control (n = 11) and *Lhx2-Cre:Rptor$^{f/f}$* (n = 8) mice at P9 and the spatial properties of RGC mosaics in the DT and VN domains were determined. (A – D) Histogram frequency plots of Voronoi domain areas (μm^2) in control (A – B) and mutant (C – D) mice reveal comparable mean area values in addition to a normal distribution of cell territories in the DT and VN domains of wild type animals and in the VN region of *Rptor*-ablated mice (A – C). In contrast, a significantly reduced mean area value and a right-skewed distribution of Voronoi domain areas were present in the DT domain of *Lhx2-Cre:Rptor$^{f/f}$* animals (D). (E – H) Histogram frequency plots of nearest neighbour distances (μm) in control (E – F) and

Biology Open • Supplementary information

mutant (G – H) mice reveal comparable mean distance values in addition to a normal distribution of RGC somata in the DT and VN domains of wild type animals and in the VN region of *Rptor*-ablated mice (E – G). In contrast, a significantly reduced mean distance value and a right-skewed distribution of nearest neighbour distance were present in the DT domain of *Lhx2-Cre:Rptor$^{f/f}$* animals (H). The VDRI and NNRI were calculated for each domain and both control and *Lhx2-Cre:Rptor$^{f/f}$* mice exhibited similar values. Abbreviations: DT, dorsotemporal; *n*, number; NNRI, nearest neighbour regularity index; P, postnatal; RGCs, retinal ganglion cells; SD, standard deviation; VN, ventronasal; VDRI, Voronoi domain regularity index.

Biology Open • Supplementary information

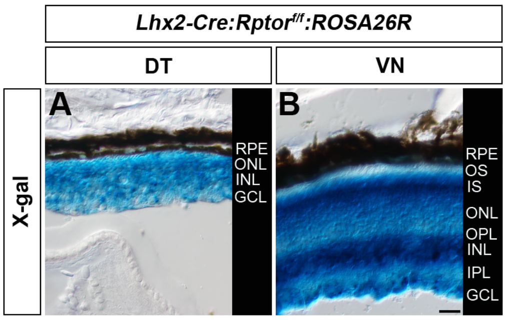

Figure S11. Lineage tracing analysis of Cre-recombinase expression in adult *Lhx2-Cre:Rptor$^{f/f}$*:R*OSA26R* mice. (A – B) Representative coronal eye sections from enucleated eyes harvested from *Lhx2-Cre:Rptor$^{f/f}$:ROSA26R* mice (*n* = 2) at 6 weeks of age demonstrates widespread X-gal staining throughout all three nuclear layers (ONL, INL and GCL) in both the DT (A) and VN (B) regions. Scale bar: (A – B) 100 µm. Abbreviations: GCL, ganglion cell layer; INL, inner nuclear layer; IPL, inner plexiform layer; IS, inner segments; ONL, outer nuclear layer; OPL, outer plexiform layer; OS, outer segments; RPE, retinal pigment epithelium.

Biology Open • Supplementary information

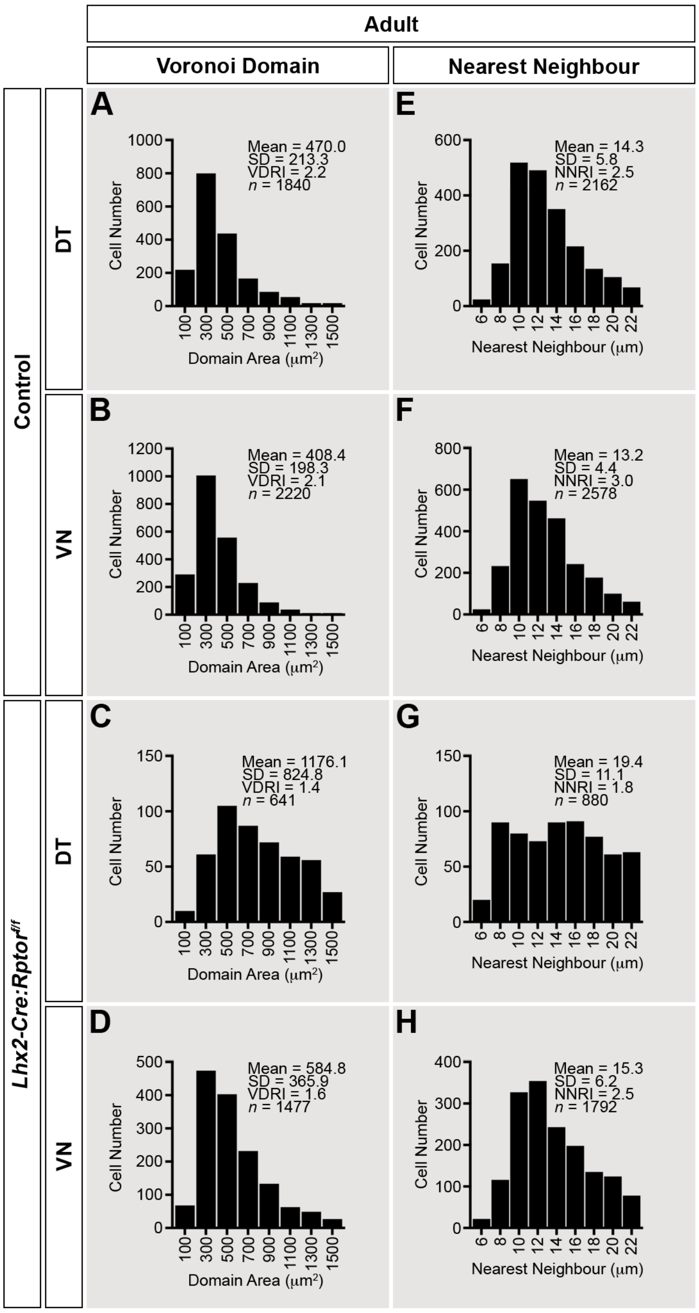

Figure S12. Histogram frequency plots of RGC mosaics in control and *Lhx2-Cre:Rptor*$^{f/f}$ adult mice. (A – H) Retinae were harvested from control (*n* = 8) and *Lhx2-Cre:Rptor*$^{f/f}$ (*n* = 8) mice at 7 weeks of age and the spatial properties of RGC mosaics in the DT and VN domains were determined. (A – D) Histogram frequency plots of Voronoi domain areas (μm^2) in control (A – B) and mutant (C – D) mice reveal comparable mean area values in addition to a normal distribution of cell territories in the DT (A) and VN (B) domains of wild type animals. In contrast, *Lhx2-Cre:Rptor*$^{f/f}$ mice exhibit significantly increased mean Voronoi domain areas in both the DT (C) and VN quadrants (D) with the DT region (C) exhibiting far greater irregularity and consequent plateau distribution when compared to the

Biology Open • Supplementary information

VN region (D). (E – H) Histogram frequency plots of nearest neighbour distances (µm) in control (E – F) and mutant (G – H) mice reveal comparable mean distance values in addition to a normal distribution of RGC somata in the DT (E) and VN (F) domains of wild type animals. In contrast, *Lhx2-Cre:Rptor*$^{f/f}$ mice exhibit significantly increased mean distance values in both the DT (G) and VN quadrants (H) with the DT region (G) exhibiting far greater irregularity and consequent plateau distribution when compared to the VN region (H). The greater irregularity in the DT retina of *Lhx2-Cre:Rptor*$^{f/f}$ mice was confirmed by differences in both the VDRI and NNRI. Abbreviations: DT, dorsotemporal; *n*, number; NNRI, nearest neighbour regularity index; RGCs, retinal ganglion cells; SD, standard deviation; VN, ventronasal; VDRI, Voronoi domain regularity index.

Biology Open • Supplementary information

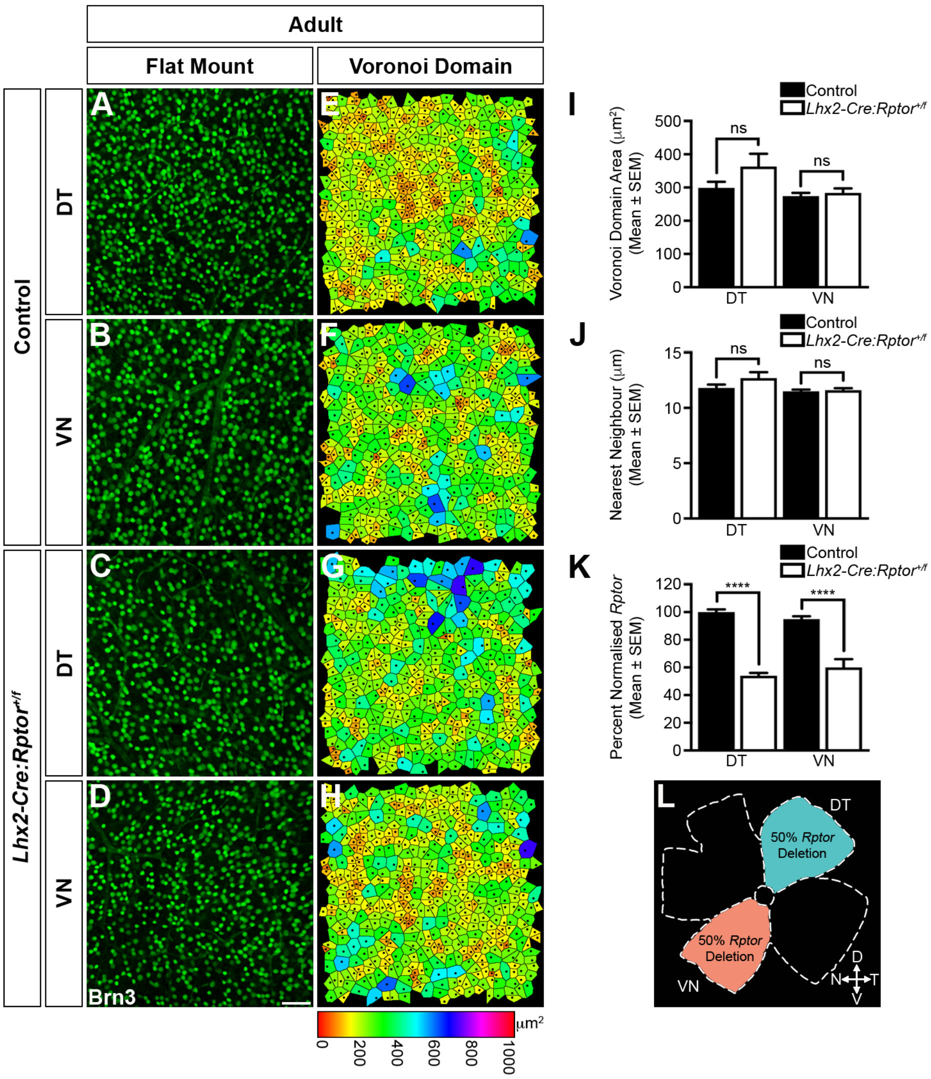

Figure S13. Heterozygous *Rptor* adult mice exhibit RGC mosaics that are comparable to control littermates. Retinae were harvested from control (n = 4) and *Lhx2-Cre:Rptor*$^{+/f}$ (n = 5) mice at 7 weeks of age and the mosaic arrangement of RGCs in the DT and VN regions (L) was determined. (A – D) Representative flat-mount images of control and *Lhx2-Cre:Rptor*$^{+/f}$ retinae demonstrate that the RGCs in both the DT and VN quadrants are arranged in comparable arrays. (E – H) The spatial properties of the RGC mosaics were determined by Voronoi domain and nearest neighbour analyses. Representative heat map diagrams reveal similar Voronoi domain areas and nearest neighbour distances within *Lhx2-Cre:Rptor*$^{+/f}$ and wild type littermates. (I – J) Quantitative analyses of the Voronoi domain (I) and nearest neighbour distances (J) demonstrate that RGCs within the DT and VN regions of *Lhx2-Cre:Rptor*$^{+/f}$ and control mice exhibit comparable values. The corresponding Voronoi domain and nearest neighbour distance histogram plots are presented in Fig. S14. (K) qPCR analysis of normalised *Rptor* levels in the DT and VN retinae of control (n = 6) and *Lhx2-*

Biology Open • Supplementary information

Cre:Rptor$^{+/f}$ (*n* = 5) mice at 6 weeks of age. Heterozygote animals exhibit a domain-specific pattern of *Rptor* recombination with approximately a 50% deletion rate being observed in both regions. (L) Schematic diagram detailing the retinal quadrants imaged for the Voronoi domain and nearest neighbour analyses. All data represents the mean ± SEM. Statistical differences were calculated using unpaired two-tailed Student's t-tests. p values are denoted as follows: ****p ≤ 0.0001. Scale bar: (A –D) 50 μm. Abbreviations: D, dorsal; DT, dorsotemporal; N, nasal; T, temporal; V, ventral; VN, ventronasal.

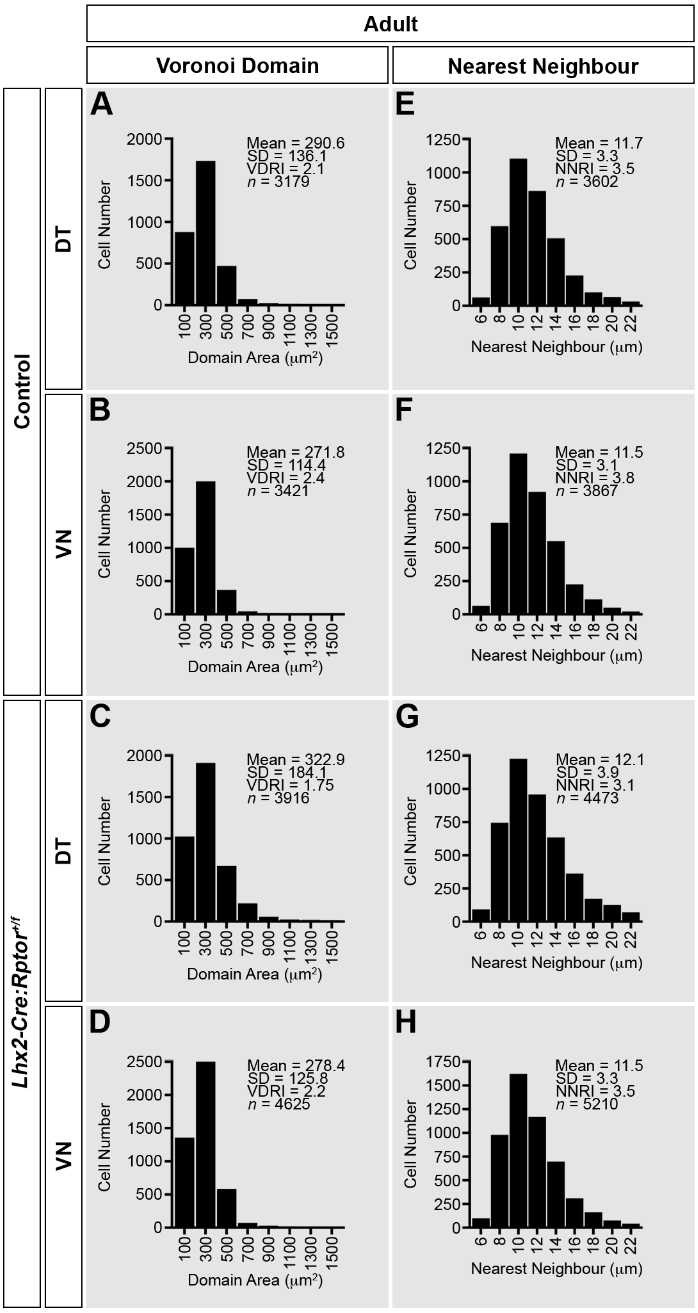

Figure S14. Histogram frequency plots of RGC mosaics in control and heterozygous *Rptor* adult mice. (A – H) Retinae were harvested from control (n = 4) and *Lhx2-Cre:Rptor*$^{+/f}$ (n = 5) mice at 7 weeks of age and the spatial properties of RGC mosaics in the DT and VN domains were determined. (A – D) Histogram frequency plots of Voronoi domain areas (μm^2) in control (A – B) and heterozygote mice (C – D) reveal comparable mean area values in addition to a normal distribution of cell territories. (E – H) Histogram frequency plots of nearest neighbour distances (μm) in control (E – F) and heterozygous mice (G – H) reveal comparable mean distance values in addition to a normal distribution of RGC somata. The observed uniformity of the RGC mosaics in both control and *Lhx2-Cre:Rptor*$^{+/f}$

Biology Open • Supplementary information

mice was confirmed by similar VDRI and NNRI values. Abbreviations: DT, dorsotemporal; *n*, number; NNRI, nearest neighbour regularity index; RGCs, retinal ganglion cells; SD, standard deviation; VN, ventronasal; VDRI, Voronoi domain regularity index.

Biology Open • Supplementary information

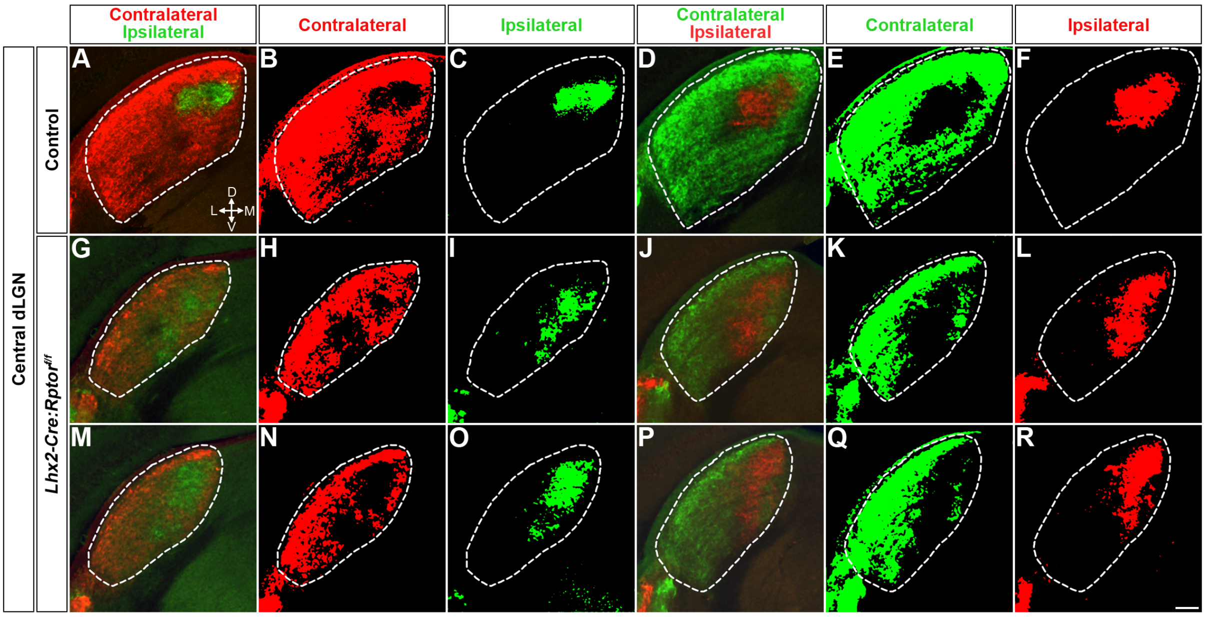

Figure S15. Aberrant retinogeniculate topography in *Lhx2-Cre:Rptor$^{f/f}$* mice. Contralateral and ipsilateral projection patterns in the central dLGN of control (n = 8) and *Lhx2-Cre:Rptor$^{f/f}$* (n = 8) mice were visualised by intraocular injections of fluorescently labelled CTB at 6 weeks of age. (A – F) Control animals exhibited characteristic contralateral and ipsilateral territories within the central dLGN. (G – R) In contrast, *Lhx2-Cre:Rptor$^{f/f}$* mice displayed varied contralateral termination topographies (H, K, N and Q) with portions of the central dLGN being largely unoccupied. Moreover, the dorsomedial ipsilateral projection was variable in position and surrounded by additional ectopic arbors (I, L, O and R). The contralateral and ipsilateral panels are presented as binarised images to allow better visualisation of the termination topographies. Dashed lines define the border of the dLGN in all images. Scale bar: (A – R) 100 µm. Abbreviations: D, dorsal; L, lateral; M, medial; V, ventral.

Biology Open • Supplementary information

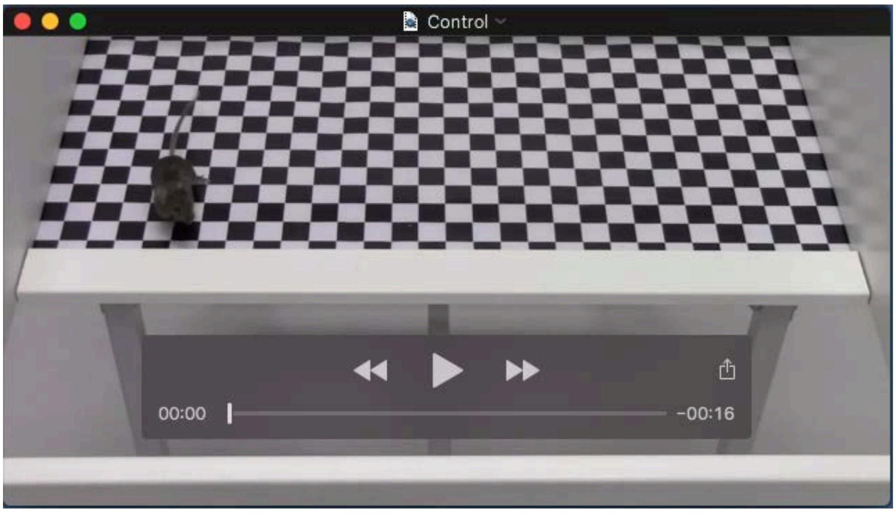

Movie 1. Typical behaviour of a control mouse while performing the visual cliff test. In this example the control animal approaches the central dividing beam and inspects the cliff but then retreats to the ground side of the testing arena.

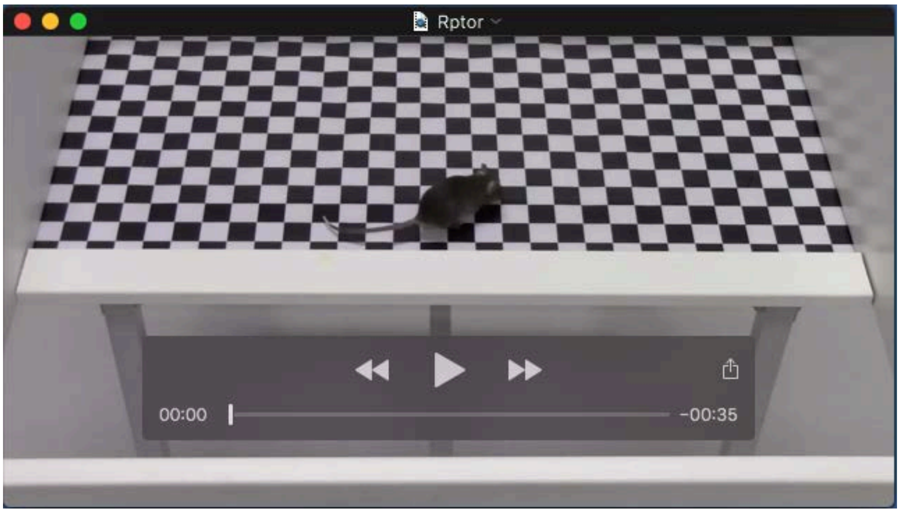

Movie 2. Typical behaviour of an *Lhx2-Cre:Rptor*$^{f/f}$ mouse while performing the visual cliff test. In this example the mutant animal approaches to the central dividing beam and inspects the cliff before crossing onto the cliff side without hesitation. The animal then returns onto the central dividing beam prior to moving to the ground side of the testing arena.

Biology Open • Supplementary information
